# Understanding speech and language in *KIF1A*-associated neurological disorder

**DOI:** 10.1038/s41431-025-01867-0

**Published:** 2025-05-16

**Authors:** Lottie D. Morison, Adam P. Vogel, John Christodoulou, Wendy A. Gold, Dylan Verden, Wendy K. Chung, Ruth Braden, Joanna Bredebusch, Simranpreet Kaur, Ingrid E. Scheffer, Angela T. Morgan

**Affiliations:** 1 Speech and Language Team, Murdoch Children’s Research Institute, Parkville, VIC Australia; 2 Department of Audiology and Speech Pathology, The University of Melbourne, Parkville, VIC Australia; 3 Redenlab Pty Ltd, Melbourne, VIC Australia; 4 Brain and Mitochondrial Group, Genomic Medicine, Murdoch Children’s Research Institute, Parkville, VIC Australia; 5 Department of Paediatrics, The University of Melbourne, Parkville, VIC Australia; 6 School of Medical Sciences, Faculty of Medicine Health, The University of Sydney, Sydney, NSW Australia; 7 Kids Neuroscience Centre, Kids Research, Children’s Hospital at Westmead, Westmead, NSW Australia; 8 Molecular Neurobiology Research Laboratory, Kids Research, Children’s Hospital at Westmead and the Children’s Medical Research Institute, Westmead, NSW Australia; 9 KIFA.org, San Francisco, CA USA; 10 Department of Paediatrics, Boston Children’s Hospital, Harvard Medical School, Boston, MA USA; 11 Department of Paediatrics, University of Melbourne, and Department of Neurology, Royal Children’s Hospital, Parkville, VIC Australia; 12 Department of Medicine, University of Melbourne, Austin Health, Heidelberg, VIC Australia; 13 Murdoch Children’s Research Institute and Florey Institute of Neuroscience and Mental Health, Parkville, VIC Australia

**Keywords:** Neurodevelopmental disorders, Epilepsy, Movement disorders, Paediatric neurological disorders, Neurodegenerative diseases

## Abstract

*KIF1A*-associated neurological disorder (KAND) is a genetic condition characterised by motor, cognitive and ophthalmologic features. The speech and language phenotype have not been systematically analysed. Here, we assess speech and language using observer- and clinician-reported outcomes, and performance outcome measures. 44 individuals (25 female) with KAND (median age 7 years, range 1–60 years) participated. Median age at diagnosis was 4 years (range 0.5–58 years). *KIF1A* variants were missense (41/44 individuals, 93%), intragenic deletion (2/44, 5%) and splice site (1/44, 2%). Age at first words was delayed (>12 months) in 38/44 (86%) individuals. At assessment, 28/44 (64%) combined words into sentences and all of the 20 individuals assessed had dysarthria. Apraxic speech features and phonological impairments occurred in children aged under 8 years. 36/37 (97%) participants had language impairment, with expressive language skills stronger than receptive (*p* = 0.02) and written (*p* = 0.03) language on the Vineland Adaptive Behaviour Scales. 7/32 (22%) caregivers reported speech and language regression. Mild to severe intellectual disability occurred in 31/33 (94%) individuals. 22/44 (50%) participants had used augmentative and alternative communication, such as key word sign or speech generating devices. Individuals had average social motivation skills in contrast to moderately impaired social cognition, communication and awareness on the Social Responsiveness Scale (*p* < 0.05). 16/44 (36%) had epilepsy and 40/44 (91%) had visual impairment, namely nystagmus (16/44, 36%), optic nerve atrophy and strabismus (both 12/44, 27%). Individuals with KAND frequently have speech and language disorders necessitating early and targeted speech and language interventions.

## Introduction

*KIF1A*-associated neurological disorder (KAND, OMIM 601255) is a rare, neurodevelopmental condition. KAND characteristics include motor disorders, intellectual disability, visual impairment, cerebral and cerebellar atrophy, seizures and speech and language disorders [1–3]. Adaptive behaviour skills decline over time particularly in those with seizures and brain structural abnormalities [3].

KAND is caused by pathogenic or likely pathogenic variants in *KIF1A* which encodes the evolutionarily conserved axonal motor protein KIF1A (kinesin family member 1a), that transports synaptic-vesicle precursors (HGNC:888) [4–6]. Pathogenic *KIF1A* variants affect axonal transport, with loss-of-function variants reducing axonal microtubule association, rate, or distance. KAND is associated with both dominant and recessive patterns [3].

Previously, KAND phenotypes were divided into distinct conditions; hereditary sensory and autonomic neuropathy 2, hereditary spastic paraplegia type 30, neurodegeneration and spasticity with or without cerebellar atrophy or cortical visual impairment syndrome, and progressive encephalopathy with oedema, hypsarrhythmia and optic atrophy syndrome [4, 7–9]. KAND phenotypes were reclassified to describe the phenotypic spectrum; i) simple KAND with spasticity in lower extremities, ataxia and peripheral neuropathy, ii) complex KAND with intellectual disability, seizures, optic nerve atrophy and cerebellar atrophy, and iii) hereditary sensory and autonomic neuropathy KAND with variants in the alternatively spliced exon [1]. Although most recently, use of these classifications has been reconsidered given that many individuals do not meet all features of a given classification [3]. Some individuals with complex KAND have childhood dementia [10, 11].

Gross motor impairment is core to KAND including hyperkinesis, mixed muscle tone (hyper, hypo, and dystonia) and ataxia [1, 3, 12]. Motor impairment is also reflected in the speech motor disorder, dysarthria, a core feature of KAND [3, 11, 13–20].

Speech and language in individuals with KAND have not been systematically characterised. Speech relates to the production and perception of speech sounds, whereas language is symbolic representation of meaning through vocabulary and grammar and includes both expressive language (output) and receptive language (understanding, input). Language skills have only been characterised via caregiver administered assessment, with abilities ranging from average performance to severely impaired language skills [1, 2]. Speech and language impairment complicated the detection of cognitive and sensory symptoms such as impaired vision and sensory neuropathy, whose incidence may delay access to care or treatment options [1–3, 11].

Some individuals with KAND use little to no speech, necessitating alternative communication methods, such as augmentative and alternative communication (AAC, e.g., key word sign, speech generating devices) [13, 19, 21]. There is a dearth of information on use of AAC in individuals in KAND [22–24].

Disease modifying treatments in KAND, such as antisense oligonucleotide (ASO) therapy, are emerging, with a recent report of the first child to receive ASO therapy [20]. The child’s gross motor skills and seizures improved, whilst cognitive performance remained stable. Speech quality was also reported to improve, including longer utterances and reduced dysarthria severity.

Lastly, progressive motor impairment and optic nerve atrophy are core features of disease progression in KAND [1–3, 12, 14]. Progressive gross motor symptoms suggest that motor speech symptoms may worsen across the disease course [25].

Characterising speech and language in individuals with KAND will inform speech and language interventions, and how best to sensitively measure speech and language changes in response to disease progression and disease modifying treatments.

## Methods

### Participants

Participants were aged >6 months. All families provided reports documenting heterozygous (likely) *KIF1A* pathogenic variants. Study materials were provided in several languages (Table 1). Caregiver assessments were completed online. All procedures were in accordance with the 1975 Declaration of Helsinki [26].

**Table 1:** Assessment battery.

Area assessed	Measure	Languages available	Age (yrs)	Skill requirement	Outcome measure type^b^	*N* completed
Medical	Medical questionnaire	English, French, Italian, Spanish, German	All	All	ObsRO	44
Feeding	ChOMPS	English	≤7	Oral feeding	ObsRO	30
Speech	Perceptual speech assessment via video call	English	All	Verbal	ClinRO	20
	Acoustic speech battery via video call	English	All	Verbal	PerfO	13
	ICS	English, French, Italian, Spanish, German	All	All	ClinRO	44
Language	Vineland-3	English, Spanish	All	All	ObsRO	37
	Vineland-2	French	All	All	ObsRO	4
	CCC-2	English	≥4	Verbal	ObsRO	18
Non-verbal communication and AAC	Communication Matrix	English, French, Italian, Spanish, German	All	Minimally verbal	ObsRO	19
	AAC questionnaire	English, French, Italian, Spanish, German	All	All	ObsRO	44
Social communication	SRS-2	English	≥2.5	All	ObsRO	30
Adaptive behaviour and motor^a^	Vineland-3	English, Spanish	All	All	ObsRO	37
	Vineland-2	French	All	All	ObsRO	4
Development, education and therapy	Medical questionnaire	English, French, Italian, Spanish, German	All	All	ObsRO	44

*AAC* augmentative and alternative communication, *CCC-2* Children’s communication checklist 2nd edition, *ChOMPS* Child oral motor proficiency scale, *ClinRO* Clinician-reported outcome, *ICS* Intelligibility in context scale, *Minimally verbal* few or no spoken words, *ObsRO* Observer-reported outcome, *PerfO* Performance outcome, *SRS-2* Social responsiveness scale 2nd edition, *Verbal* uses spoken sentences, *Vineland-3* Vineland adaptive behaviour scales 3rd edition comprehensive caregiver form, *Vineland-2* Vineland adaptive behaviour scales 2nd edition comprehensive caregiver form, *yrs* years.

^a^An overall adaptive behaviour composite (ABC) score is derived from domain scores (Vineland-3 and ≥7 years Vineland-2: communication, daily living, socialisation; Vineland-2: <7 years: communication, daily living, socialisation, motor). Daily living and socialisation domains have three subdomains (daily living: self-care, domestic and community living skills; socialisation: interpersonal, play and leisure and adaptive behaviour). Motor skill has two subdomains (fine and gross motor). Vineland-3 assesses motor skills up to 9 years, 11 months, whereas Vineland-2 assesses motor skills up to 6 years, 11 months.

^b^US Food and Drug Administration (FDA). (2022). Patient-focused drug development: selecting, developing, or modifying fit-for purpose clinical outcome assessments. Access 2nd August 2024. Available at: https://www.fda.gov/regulatory-information/search-fda-guidance-documents/patient-focused-drug-development-selecting-developing-or-modifying-fit-purpose-clinical-outcome.

### Development and past medical history

Caregivers, including parents and one sibling, completed a comprehensive developmental and medical questionnaire including medical conditions, developmental milestones, regression, education, therapy, interests, strengths and dislikes (Table 1) [22–24, 27]. English-speaking families were interviewed, and clinician reports were reviewed. Caregivers provided reports of standardised cognitive testing from their child’s treating clinician.

### Feeding

The Child Oral Motor Proficiency Scale (ChOMPS) assessed feeding (normative data used for ≤7years, 7-year old data for those >7 years) [28]. ChOMPS has four domains, combined to provide a total score: complex movement patterns (e.g., licking food off lip), basic movement patterns (e.g., holding a toy), oral motor coordination (e.g., moving jaw to chew), and fundamental oral motor skills (e.g., closing lips). High ChOMPS scores indicate stronger feeding skills; scores >10%ile indicate no feeding concerns.

### Speech

‘Verbal’ participants were defined as being able to combine words to form spoken phrases or sentences. ‘Minimally verbal’ participants had few or no spoken words or were not combining words.

Speech pathologist (LM) assessed English-speaking participants’ speech over video call, eliciting speech samples from a 5-min conversation, single word test and syllable repetition (diadochokinesis) tasks. Speech subsystems were rated; respiration, phonation, resonance, articulation, and prosody, to assess for dysarthria (motor speech disorder) [29]. Oral motor assessment informed differential diagnosis of speech disorders. The American Speech Language and Hearing Association’s diagnostic criteria were used to classify childhood apraxia of speech (CAS) [30, 31]. Phonological delay (typical albeit age-inappropriate speech sound errors) and disorder (atypical errors) were diagnosed [32]. An articulation disorder was indicated in the instance of absent or distorted speech sounds. The Intelligibility in Context Scale (ICS) rated intelligibility from never understood (1) to always understood (5) by communication partners [33].

Objective speech features were derived from an online acoustic analysis using Redenlab’s® Analyze pipeline (Supplementary Table 1) [34]. A control group consisted of two age and sex-matched participants for each participant with KAND and audio data. Control participants self-referred and had no pre-existing medical or developmental conditions which could impact speech or language (e.g., hearing loss).

### Language and AAC

Comprehensive caregiver forms from the Vineland Adaptive Behaviour Scales were administered (Vineland-3: English and Spanish; Vineland-2: French) [35, 36]. The Vineland communication domain (normative mean = 100, SD = 15) assessed language skills across receptive, expressive and written language subdomains (normative mean = 15, SD = 3).

English-speaking caregivers of verbal participants completed the Children’s Communication Checklist Second Edition (CCC-2) [37]. The CCC-2 assesses ten scales: speech, syntax, semantics, coherence, initiation, stereotyped language, use of context, nonverbal communication, social and interests (normative mean = 10, SD = 3).

The Communication Matrix assessed minimally verbal participants’ communication behaviours from 1 (pre-intentional behaviours interpreted by communication partners) to 7 (combining spoken words, signs or symbols) across the communication functions of i) requesting, ii) refusing, iii) social communication and iv) communicating for information [38].

Caregivers provided information on AAC use and perceptions via questionnaire.

### Social communication

To look for features of autism spectrum disorder, we used the Social Responsiveness Scale Second Edition (SRS-2) to assess social communication and restricted and repetitive behaviours, key autism diagnostic criteria [39, 40]. SRS-2 social communication domain includes social awareness, cognition, communication and motivation subscales. The social communication and restricted and repetitive behaviour domains provide a total T-score. T-scores ≤59 are within normal limits, 60–65 mild, 66–75 moderate and >76 severely impaired.

### Adaptive behaviour and motor skills

Vineland-3 and Vineland-2 assessed communication, daily living, socialisation and motor skill domains [35, 36]. Communication, daily living and socialisation domains combine to create an adaptive behaviour composite score.

### Statistical analysis

Non-parametric measures were used due to small cohort size and non-normative distribution. A Wilcoxon-signed rank test compared receptive, expressive, and written language skills. Mann–Whitney U tests analysed differences between control and KAND participants’ age and acoustic analysis measures. Spearman’s correlation co-efficient assessed the relationship between age and average Intelligibility in Context score, SRS-2 total T-score, and Vineland-3 scores. Friedman tests compared Vineland-3 domains, SRS-2 subscales, and CCC-2 scales, and post-hoc Dunn’s multiple comparisons tests were corrected for multiple comparisons by statistical hypothesis testing. Fisher’s exact tests examined the association between epilepsy, and intellectual disability and Magnetic Resonance Imaging (MRI) abnormalities.

## Results

### Participants

44 individuals (25 female) with KAND participated from 13 countries (United States *n* = 15, Australia *n* = 7, France *n* = 6, United Kingdom *n* = 4, Spain, Italy and Canada (all *n* = 2), Chile, Colombia, German, Japan, New Zealand and Sweden (all *n* = 1)). Median age of diagnosis was 4 years (range 0.5–58 years, Supplementary Table 2). Median age was 7 years, 11 months (range 1–60 years, Table 2). Participant ID5 died at age 18 months in the year prior to the study. Caregivers who completed study information were mothers (38/44, 86%), fathers (5/44, 11%) and a sister (1/44, 2%).

**Table 2:** Speech and language skills of 44 individuals with *KIF1A*-associated neurological disorder.

Participant ID	Age at assessment (range, yrs)	Protein change	Milestones		Speech		Language^b^			
		(NP_001230937.1)	First words (mo)	Short sentences (yrs)	Verbal or minimally verbal^a^	Motor speech disorder	Overall language^c^ (mean = 54, SD = 22)	Receptive^d^ (mean = 7, SD = 4)	Expressive^d^ (mean = 8, SD = 5)	Written^d^ (mean = 7, SD = 4)
1	11–12	p.(Arg11Gln)	22	2–3	Verbal	Dysarthria	Low	Low	Low	Low
2	39–40	p.(Arg11Gln)	15–18	2–3	Verbal	Dysarthria	Mod	Average	Average	Mod
3	17–18	p.(Arg11Gln)	<12	2–3	Verbal	Dysarthria	Average	Average	Average	Average
4	11–12	p.(Gly97Arg)	12–15	2–3	Verbal	Dysarthria	Mod	Mod	Mod	Low
5	1–2	p.(Thr99Lys)	NYA	NYA	Minimally verbal	NA	Low	Low	Low	NA
6	11–12	p.(Thr99Met)	12–15	NYA	Minimally verbal	NA	NA	NA	NA	NA
7	3–4	p.(Gly102Ser)	12–15	NYA	Minimally verbal	NA	Low^e^	Low	Low	Mod
8	13–14	p.(Gly102Ser)	<12	2–3	Verbal	NA	Mod	Average	Mod	Low
9	23–24	p.(Lys103Thr)	12–15	9	Verbal	Dysarthria	Low	Low	Low	Low
10	15–16	p.(Gly117Val)	≥2 yrs	4–5	Verbal	Dysarthria	Mod	Mod	Mod	Low
11	1–2	p.(Glu148Gly)	NYA	NYA	Minimally verbal	NA	Low^e^	Low	Low	NA
12	23–24	p.(Glu148Val)	NYA	NYA	Minimally verbal	NA	Low	Low	Low	Low
13	21–32	p.(Leu157His)	12–15	10	Verbal	NA	Low	Low	Low	Low
14	13–14	p.(Arg169Lys)	12–15	2–3	Verbal	Dysarthria	Mod	Mod	Average	Low
15	1–2	p.(Gly199Glu)	NYA	NYA	Minimally verbal	NA	Low	Low	Low	NA
16	1–2	p.(Ala202Pro)	NYA	NYA	Minimally verbal	NA	Mod	Mod	Mod	NA
17	1–2	p.(Ser214Asn)	<12	NYA	Minimally verbal	NA	Low	Low	Low	NA
18	11–12	p.(Arg216His)	<12	6–7	Verbal	Dysarthria	Mod	Low	Average	Low
19	3–4	p.(Arg216His)	12–15	4–5	Verbal	Dysarthria, CAS	Low	Low	Low	Mod
20	3–4	p.(Arg216His)	12–15	NYA	Minimally verbal	NA	Mod	Mod	Low	Mod
21	3–4	p.(Leu249Pro)	12–15	NYA	Minimally verbal	NA	NA	NA	NA	NA
22	23–24	p.(Leu249Pro)	4–5yrs	20	Verbal	Dysarthria	Low	Mod	Low	Low
23	59–60	p.(Arg254Gln)	≥18	4–5	Verbal	Dysarthria	Low	Low	Average	Low
24	5–6	p.(Arg254Trp)	3 yrs	4–5	Verbal	Dysarthria	Low	Low	Low	Low
25	3–4	p.(Arg254Trp)	2 yrs	4–5	Verbal	Dysarthria, CAS	Low	Low	Low	Mod
26	3–4	p.(Arg254Trp)	<12	2–3	Verbal	Dysarthria	Low	Low	Low	Mod
27	3–4	p.(Ile271Thr)	NYA	NYA	Minimally verbal	NA	Low	Low	Low	Low
28	19–20	p.(Lys280Arg)	15–18	4–5	Verbal	NA	Mod	Average	Average	Mod
29	7–8	p.(Arg307Gln)	NYA	NYA	Minimally verbal	NA	NA	NA	NA	NA
30	3–4	p.(Arg307Gln)	NYA	NYA	Minimally verbal	NA	Low	Low	Low	NA
31	7–8	p.(Arg307Gln)	12–15	4–5	Verbal	Dysarthria	Low	Low	Low	Low
32	1–2	p.(Arg307Gln)	NYA	NYA	Minimally verbal	NA	Low	Low	Low	NA
33	5–6	p.(Arg316Trp)	NYA	NYA	Minimally verbal	NA	Low	Low	Low	Low
34	11–12	p.(Arg316Trp)	30	4–5	Verbal	Dysarthria	Low	Low	Mod	Low
35	11–12	p.(Arg316Trp)	4 yrs	6–7	Verbal	Dysarthria	Low	Low	Mod	Low
36	3–4	p.(Arg316Trp)	<12	2–3	Verbal	Dysarthria	Mod	Low	Mod	Mod
37	3–4	p.(Arg316Trp)	12–15	2–3	Verbal	NA	Low	Low	Mod	Low
38	13–14	p.(Arg316Trp)	2 yrs	4–5	Verbal	Dysarthria	Mod	Average	Average	Low
39	3–4	p.(Arg316Trp)	NYA	NYA	Minimally verbal	NA	Low	Low	Low	Mod
40	9–10	p.(Ala326Pro)	15–18	6–7	Verbal	NA	Mod	Mod	Mod	Low
41	7–8	p.(Gln671Pro)	15–18	4–5	Verbal	Dysarthria	Mod	Average	Mod	Low
42	9–10	p.(Ser49_Tyr56delinsAsn)	≥18	≥8	Verbal	NA	Low^e^	Low	Low	Low
43	17–18	p.(Ser49_Tyr56delinsAsn)	≥18	6–7	Verbal	NA	Low^e^	Mod	Low	Low
44	3–4	c.1038-1G>A	≥18	4-5	Verbal	NA	Mod	Low	Mod	Low

^a^Verbal individual used spoken sentences to communicate.

^b^Vineland adaptive behaviour scales third edition communication domain and subdomains.

^c^Communication domain: Average (>85), Mod (moderately low 71–85), Low (20–70).

^d^Communication subdomains: Average (>12), Mod (Moderately low 10–12), Low (1–9) Written language not assessed for children <3 years.

^e^Vineland adaptive behaviour scales second edition, *Mo* Months, *NA* Not assessed, *NYA* Not yet achieved, *Yrs* Years.

**Fig. 1:**
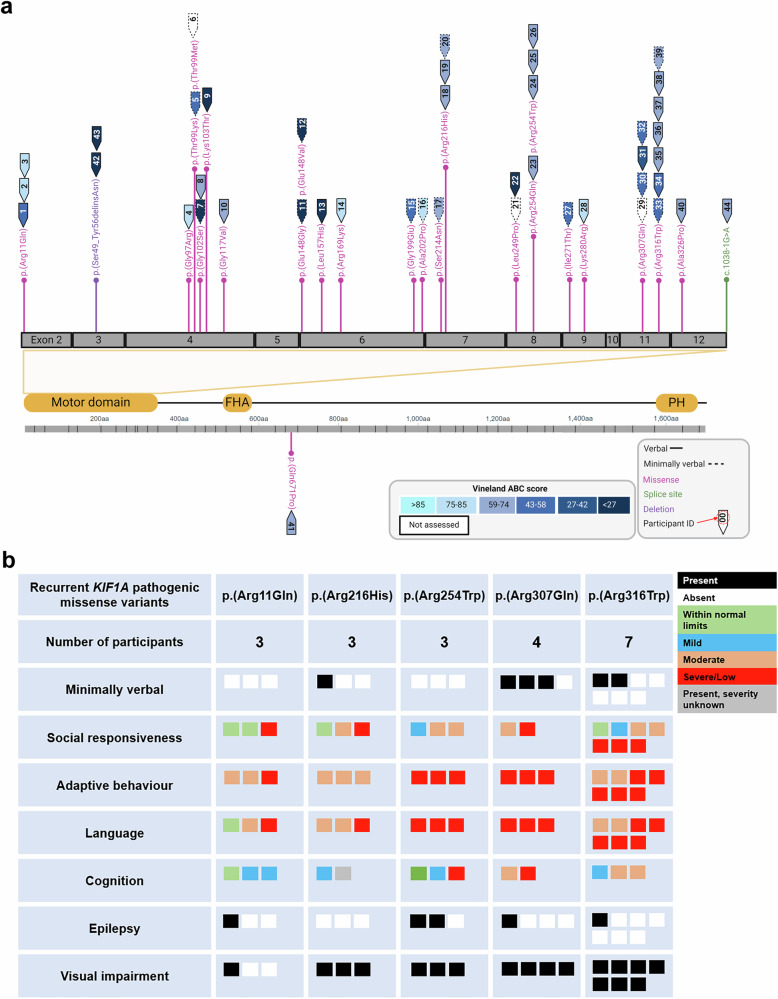
***KIF1A*** variants in this cohort. **a** Variants and adaptive behaviour of 44 individuals with *KIF1A*-associated neurological disorder. Adaptive behaviour composite scores (normative mean=100, SD = 15) from the Vineland Adaptive Behaviour 2^nd^ (*n* = 4) and 3^rd^ (*n* = 37) edition colour coded by severity as per figure legend. Verbal participants (solid line, *n* = 28) combined words into spoken sentences, and minimally verbal participants (dashed line, *n* = 16) had few or no spoken words. **b** Phenotype of participants with *KIF1A*-associated neurological disorder with recurrent *KIF1A* pathogenic missense variants in this cohort. Minimally verbal participants had few or no spoken words, and verbal participants combined words into spoken sentences. Social responsiveness assessed by the Social Responsiveness Scale 2nd Edition: within normal limits <60, mild 60–65, moderate 66–75, severe >76. Cognition assessed by treating clinicians, intelligence quotient: Average (within normal limits, including borderline) (>70), Mild [50–70], Moderate [35–50], Severe (<35). Language and adaptive behaviour composite assessed by Vineland Adaptive Behaviour Scales 3rd edition: Average (within normal limits >85), Moderate (moderately low 71–85), Low [20–70]. Epilepsy and visual impairment assessed by caregiver report.

30/44 (68%) participants had de novo variants and 2/44 (5%) variants were inherited from fathers who were mosaic for their child’s pathogenic variant. Inheritance was unknown in 12/44 (27%). 41/44 (93%) participants had missense variants, 40 of which were in the *KIF1A* motor domain (Fig. 1a). Two siblings had a deletion p.(Ser49_Tyr56delinsAsn), and one participant had a splice site variant c.1038-1G>A. Five recurrent missense variants were shared by >2 participants (Fig. 1b).

### Development

Speech and language milestones were delayed (Table 2). Few participants (6/44, 14%) said their first words by 12 months, with 11/44 (25%) at 12–15 months, 4/44 (9%) at 15–18 months, and 12/44 (27%) at ≥18 months; the oldest age was 5 years. 3/11 (27%) of participants who had not said their first words were >4 years of age. Age at combining spoken words into sentences was similarly delayed for participants ≥2 years; 9/41 (22%) at 2–3 years, 11/41 (27%) at 4–5 years, 4/41 (10%) at 5–7 years, and 4/41 (10%) at ≥8 years. 13/41 (32%) participants were not yet combining words. ID12 was not yet combing words in adulthood (1/7, 14% of adult participants).

Motor milestones were severely affected; 28/44 (64%) individuals did not walk independently (range 1–31 years old, Supplementary Table 3). Of those who could walk independently (16/44, 36%), 14 walked after 16 months of age.

No participants were receiving ASO therapy. 41/44 (93%) participants had occupational therapy and 42/44 (95%) had physiotherapy. At time of study, 23/44 (52%) participants were receiving occupational therapy and 29/44 (66%) physiotherapy. 38/44 (86%) participants had seen a speech pathologist. Approximately half of school age participants attended mainstream and half specialist settings (Supplementary Table 4). Participant ID2 worked part-time and had a diploma.

Of the 32/44 (73%) of caregivers who were asked about regression, 7/32 (22%) reported speech and language regression, 10/32 (31%) gross and 4/32 (13%) fine motor regression, and 1/32 (3%) had lost play skills.

31/44 (70%) caregivers reported their child’s interests, strengths and dislikes. Caregivers identified socialisation (24/31, 77%), determination (14/31, 45%) and sense of humour (12/31, 39%) as strengths. Participants’ interests were music (15/31, 48%), swimming (7/31, 23%) and playing games with others (20/31, 65%). 21/31 (68%) participants disliked loud noises.

Visual impairment was common (40/44, 91%), namely nystagmus (16/44, 36%), optic nerve atrophy (12/44, 27%), strabismus (12/44, 27%), cortical visual impairment (11/44, 25%), myopia (10/44, 23%) and hypermetropia (9/44, 20%, Supplementary Tables 3 and 5).

ID33 had mild (25-39dBHL) sensorineural hearing loss but did not use their hearing aids. 3/44 (7%) participants had tympanostomy tubes (Supplementary Table 5).

**Fig. 2:**
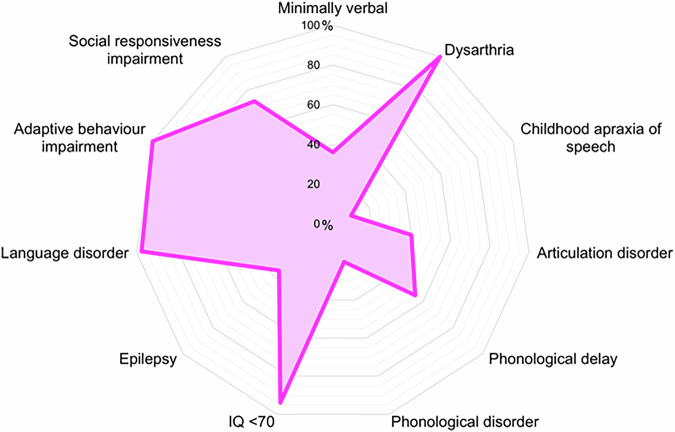
Neurodevelopmental diagnoses in this cohort of individuals with *KIF1A*-associated neurological disorder. Minimally verbal participants had few or no spoken words, and verbal participants combined words into spoken sentences (assessed *n* = 44). Dysarthria, childhood apraxia of speech, articulation disorder, phonological delay and disorder assessed via perceptual speech assessment by a speech pathologist (*n* = 20). IQ (intelligence quotient) assessed by treating clinicians (*n* = 33), scores <70 indicating an intellectual disability. Epilepsy assessed by caregiver report (*n* = 44). Language disorder and adaptive behaviour assessed by Vineland Adaptive Behaviour Scales 2^nd^ (*n* = 4) a^nd^ 3^rd^ (*n* = 37) edition, scores <86 indicating impairment. Social responsiveness assessed by the Social Responsiveness Scale 2^nd^ Edition (*n* = 30), scores >59 indicating impairment.

All participants (44/44, 100%) had developmental delay. 33/44 (75%) participants had undergone a cognitive assessment and most (31/33, 94%) had an intellectual disability ranging from mild to severe (Fig. 2, Supplementary Table 3). The two participants without intellectual disability had average (ID1, verbal intelligence quotient 50^th^ percentile) and borderline cognition (ID26, cognition 5^th^ percentile). Neurodevelopmental diagnoses also included autism spectrum disorder and attention deficit hyperactive disorder (both 12/44, 27%).

### Past medical history

Of the 43 families who provided perinatal information, 18/43 (42%) had complications (additional medical data in Supplementary Tables 3–6).

16/44 (36%) participants had epilepsy, with 6/44 (14%) having more than one seizure type and 8/16 (50%) taking multiple antiseizure medications. MRI showed abnormalities in 33/43 (77%) participants. Epilepsy was not associated with vision impairment (*p* > 0.99), intellectual disability (*p* = 0.52) or MRI abnormalities (*p* = 0.06).

Sleep disturbance occurred in 24/44 (55%) and was characterised by frequent waking (14/44, 32%), early waking (9/44, 20%), difficulty falling asleep (6/44, 14%), and sleep apnoea (2/44, 5%) (Supplementary Table 3, Supplementary Fig. 1).

Challenging behaviours in 16/44 (36%) participants included repetitive behaviour (11/44, 25%), impulsivity (10/44, 23%), hyperactivity (9/44, 20%), obsessions (9/44, 20%), anxiety (9/44, 20%) and aggression (8/44, 18%, Supplementary Table 3). 4/44 (9%) had an anxiety disorder, and 1/44 (2%) had rapid cycle bipolar disorder.

### Feeding

In infancy, children were breast (34/44, 78%) and/or bottle fed (19/44, 43%). 3/44 (7%) participants were enterally fed, with participant 5 requiring it from infancy.

Half of participants (15/30, 50%) who completed the ChOMPS were >7 years of age (median = 14.5 years, range 8–31 years) (Supplementary Fig. 2). The median age for those ≤7 years was 3 years, 5 months (range 1–7 years). Participants were often below the 5%ile for complex and basic movement patterns (≤7 years: complex 11/20, 55%, basic 7/15, 47%; >7 years: complex 9/20, 45%, basic 8/15, 53%). Oral motor coordination and fundamental oral skills were in the 5–10%ile (both 8/30, 27%). Overall, 19/30 (63%) participants had total ChOMPS scores below the 5%ile.

### Speech

28/44 (64%) participants were verbal, of whom 20/28 (71%) participants were assessed (Fig. 1, Table 2). Those who were not assessed did not speak English (6/28, 21%) or were not available (2/28, 7%).

**Fig. 3:**
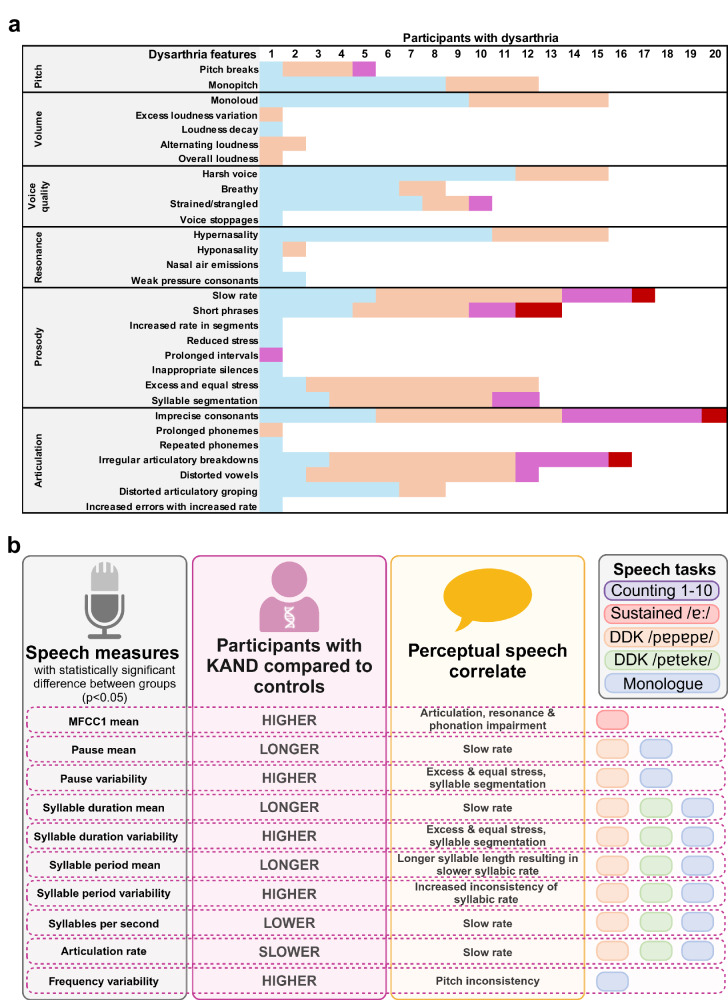
Perceptual and acoustic speech features in verbal individuals. **a** Perceptual dysarthria features of 20 participants with *KIF1A*-associated neurological disorder. Perceptual speech assessment conducted by a speech pathologist and rated using a speech subsystems. Blue = mild, orange = moderate, purple = marked, red = severe. **b** Acoustic speech characteristics in participants with *KIF1A*-associated neurological disorder and age and sex matched control participants. Analysed using Redenlab’s® Analyze pipeline. Statistically significance tested with Mann–Whitney U Test (see Supplemental results). DDK diadochokinesis, F0 fundamental frequency, MFCC mel-frequency cepstral coefficient, SD standard deviation. Counting 1–10: 11 participants with KAND, 22 control participants (no significant differences between groups), Sustained/ɐː/: 11 participants with KAND, 22 control participants, DDK/pɐtɐkɐ:/: 11 participants with KAND, 22 control participants, DDK/pɐtɐkɐ/: 10 participants with KAND, 20 control participants, Monologue: 10 participants with KAND, 20 control participants. Created in https://BioRender.com.

Dysarthria was universal (20/20, 100%) (Figs. 2, 3, Table 2). Dysarthria features included imprecise consonants (20/20, 100%), slow rate (17/20, 85%), vocal harshness (15/20, 75%), hypernasality (16/20, 80%), and monoloudness (minimal volume variation, 15/20, 75%, Fig. 3). Two children (≤4 years, 2/20, 10%) also had apraxic speech features; including inconsistent errors and distorted articulation.

7/20 (35%) participants had a phonological delay; the most common error pattern was gliding (“r” or “l” to “w” in 7/20, 35%). 4/20 (20%) participants also had a phonological disorder and were all under 8 years of age. 7/20 participants (35%) had an articulation disorder, often an interdental lisp (4/7, 57%).

Participants were rated as sometimes understood (mean = 3, SD = 1) by caregivers, family members and teachers. Participants were less intelligible to friends, acquaintances, and strangers, being rarely understood (mean = 2, SD = 1). Average intelligibility (ICS score) positively correlated with age (*p* < 0.0001, r = 0.57, CI 0.33–0.74).

13/44 (30%) English-speaking participants completed the acoustic speech battery (Supplemental Results). Measures of timing and vocal control derived from tasks with higher motoric (diadochokinesis) and cognitive load (monologue task) differed between participants and controls (Fig. 3). Participants with KAND produced longer and more variable pauses and syllable duration than controls. They also had slower syllabic and articulation rates. Objective acoustic findings reflected subjective perceptual speech features, such as slow rate, excess and equal stress, and syllable segmentation.

### Language and AAC

37/44 (84%) participants completed the Vineland-3 with overall language skills severely impaired (mean = 57, SD = 21). 22/37 (59%) participants had low language skills (20-70 standard score); only participant 3 had average language skills (>86 standard score) (Table 2). Receptive (mean = 7, SD = 5; normative mean 15) and expressive (mean = 8, SD = 5) language skills were also low. Yet, receptive and expressive skills were sometimes dissociated. Expressive language skills were stronger than receptive (W = 248, *p* = 0.02), and 6/37 (16%) individuals having average receptive and 7/37 (19%) expressive language skills. Only participant 3 had average written language skills (1/31 participants over 3 years, 3%, mean = 7, SD = 4). Written language skills were commensurate with receptive language (W = −93, *p* = 0.22) but weaker than expressive language skills (W = −210, *p* = 0.03). Age did not correlate with overall language skills (r = −0.08, CI −0.26 to 0.40, *p* = 0.64). The language skills of the 4/44 (9%) participants who completed the French Vineland-2 are in Table 2.

The CCC-2 scale scores (*n* = 18) ranged considerably between participants (Supplementary Fig. 3) and were low overall (means ranging 2.72 (use of context) to 4.9 (interests)), although some participants had average scores. CCC-2 scale scores did not correlate with one another (X^2^(9) = 20.76, *p* = 0.01). Dunn’s multiple comparisons test revealed no significant differences between CCC-2 scale scores (all *p* > 0.09).

On the Communication Matrix, only 1/19 (5%) combined spoken words (Level 7). 6/19 (32%) participants used single words or signs (Level 6). On average, participants’ highest communication level was conventional communication behaviours (Level 4, e.g., pushing/pulling, simple gestures). 4/19 (21%) participants used solely pre-intentional communicative behaviours (Level 1, e.g., crying, all ≤3 years of age). Refusal (13/19, 68%), making choices (12/19, 63%) and requesting more (11/19, 58%) were relative strengths. Most participants could not ask questions (2/19, 11%), make comments (3/19, 16%) or request an absent object (4/19, 21%).

22/44 (50%) participants had used AAC, including key word sign (10/22, 45%) and aided AAC (9/22, 41%) (Supplementary Table 4). 4/22 (18%) participants had used key word sign and aided AAC, although some were not yet using these systems independently. Aided AAC included high-tech and low-tech systems (both 4/8 50%). 9/22 (41%) participants did not specify AAC type.

15/22 (68%) caregivers of participants who used AAC agreed that their speech pathologist had adequate AAC knowledge to support their child. 13/44(30%) caregivers thought that their child would communicate more effectively by increasing their AAC use. 26/44 (59%) caregivers understood that AAC would not inhibit their child’s speech.

### Social communication

On SRS-2, social motivation was within normal limits (≤59, mean = 59, SD = 15). All other subscales (social awareness, cognition, communication, restrictive and repetitive behaviours) were moderately impaired (scores 66–75). Total social responsiveness T-score was moderately impaired (mean = 68, SD = 12); 8/30 (27%) participants had average social responsiveness, 4/30 (13%) mild (60–65), 10/30 (33%) moderate, and 8/30 (27%) severe (>76). Individuals with stronger cognitive ability were more likely to have average social responsiveness: 4/5 (80%) individuals with cognitive data and average social responsiveness had a mild intellectual disability.

Social motivation on the SRS was the only subdomain that was markedly stronger than social cognition (Z = 4.21, *p* = 0.0004), social communication (Z = 3.07, *p* = 0.032), restrictive and repetitive behaviours (Z = 5.18, *p* < 0.0001), social awareness (Z = 3, *p* = 0.04) and total score (Z = 4.21, *p* = 0.0004). Social responsiveness subscales were not associated with age (all *p* > 0.1).

### Adaptive behaviour and motor skills

**Fig. 4:**
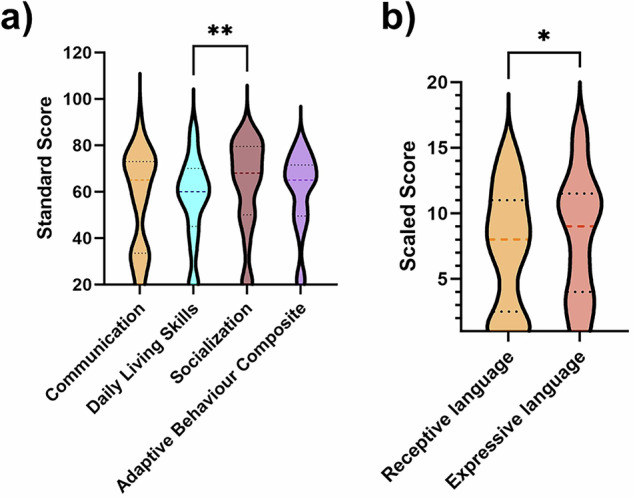
Vineland-3 scores in 37 participants with *KIF1A*-associated neurological disorder. **a** Vineland-3 domain scores in 37 participants with *KIF1A*-associated neurological disorder. Vineland Adaptive Behaviour Scales 3^rd^ Edition standard score normative mean = 100, normative SD = 15. Communication (mean = 57, SD = 21), daily living (mean = 57, SD = 18) and socialisation (mean = 63, SD = 20) skills. Overall adaptive behaviour composite score (mean = 60, SD = 17). Vineland-3 domains differed (X^2^(3) = 12.77, *p* = 0.0013); socialisation was significantly stronger than daily living skills (Z = 3.26, *p* = 0.0011). Upper dotted line = 3^rd^ quartile, lower dotted line = 1^st^ quartile, dashed middle line = median. **b** Vineland-3 communication subdomain scores in 37 participants with *KIF1A*-associated neurological disorder. Vineland Adaptive Behaviour Scales 3^rd^ Edition scaled score normative mean = 15, normative SD = 3. Receptive language (mean = 7, SD = 5) and expressive language (mean = 8, SD = 5) assessed in 37 participants. Written language skills (mean = 7, SD = 4) assessed for 31 participants >3 years. Expressive language skills were significantly stronger than receptive language skills (W = 248, *p* = 0.02).

The Vineland-3 assessed daily living and socialisation skills (Fig. 4). The group’s average daily living skills were low (mean = 57, SD = 18) with only 2/37 (5%) participants (IDs 2, 14) having average daily living skills. Vineland-3 domains differed (X^2^(3) = 12.77, *p* = 0.0013). While socialisation skills were stronger than daily living skills (mean = 63, SD = 20, Z = 3.26, *p* = 0.0011), socialisation and daily living skills subscales were low overall (means = 7–9, SD = 3–4).

Daily living skills (r = -0.05, CI −0.38 to 0.29, *p* = 0.78), socialisation (r = −0.02, CI −0.35 to 0.32, *p* = 0.91) and adaptive behaviour composite (r = 0.04, CI −0.29 to 0.37, *p* = 0.82) scores did not correlate with age.

French Vineland-2 adaptive behaviour composite scores are in Fig. 1a. No participants had an average adaptive behaviour composite score (>85, mean = 60, SD = 17), and there was no clear association between *KIF1A* variant and adaptive behaviour composite score (Fig. 1).

Vineland-3 assessed motor skills for 19 participants under 10 years of age, with motor skills being the lowest domain (mean = 47, SD = 18). 19/44 (43%) participants were not walking; 1/18 had stopped walking. Of the 25/44 (57%) who were walking, 10/44 (23%) used a mobility aid, 6/44 (14%) had an unsteady gait, and 5/44 (11%) could only walk a limited distance. Caregivers reported that 4/44 (9%) had a normal gait.

### Phenotype-genotype analysis

There was considerable phenotypic heterogeneity among the five recurrent missense variants (Fig. 1b). Participants with p.(Arg11Gln) and p.(Arg216His) pathogenic variants had a milder phenotype: they had the strongest adaptive behaviour, and 3/3 (100%) participants with p.(Arg11Gln) combined spoken words at 2-3 years, though this milestone was more variable for individuals with p.(Arg216His). Milestones were more delayed for individuals with p.(Arg254Trp): age at combining words was 4-5 years (3/3, 100%). The phenotype of individuals with p.(Arg316Trp) varied considerably across communication, social responsiveness, vision and adaptive behaviour skills. The phenotype associated with p.(Arg307Gln) was more severe: most individuals could not combine words, all had visual impairment, and all had severe language and adaptive behaviour impairment.

## Discussion

We systematically characterised speech and language in 44 individuals with KAND. We found that speech and language disorder were pervasive [1, 3]. 64% of individuals were verbal, but all had dysarthria. All but one had language impairment.

Individuals with KAND have been reported with dysarthria, yet specific dysarthric features and severity have not been quantified [13, 14, 16, 20]. In our cohort, dysarthria ranged from mild to severe, and common dysarthric features resulted from impairments across the speech systems of respiration, phonation, articulation and resonance. Dysarthric features were consistent with ataxic dysarthria, correlating with cerebellar involvement noted in KAND [1, 3]. Co-occurring speech disorders, such as phonological diagnoses and apraxia features, were also evident, particularly in younger individuals. Consequently, intelligibility was impaired, and participants were, on average, rarely understood by unfamiliar communication partners. Despite the heterogenous nature of KAND, objective acoustic analyses revealed distinct, objective motor speech impairments compared to controls, notably slower articulation rate, with longer and more variable pause length. These manifest in perceptually slow and effortful speech.

Similarly, language skills ranged from average to severely impaired, with expressive stronger than receptive skills. In autistic individuals similar patterns of relative strengths in expressive language ability are seen [41]. Cognition and literacy were also commonly impaired, with only a handful of individuals having average cognition or literacy skills.

The SRS-2 social motivation subscale, Vineland-3 socialisation domain and caregiver report emphasised individuals’ strong desire to socialise. Caregivers also noted that their children were determined and had a sense of humour. These strengths should be harnessed in therapy sessions, along with interests such as music, to support successful intervention.

In terms of genotype-phenotype analysis of the five recurrent missense pathogenic variants, the three participants with p.(Arg11Gln) of *KIF1A* had a less severe phenotype in terms of visual impairment, intellectual disability, language disorder and social responsiveness impairment.

Cohort size and few recurrent variants limited further analysis of genotype-phenotype correlations. Yet, individuals with p.(Arg216) and p.(Arg254) variants have partially retained KIF1A protein function, whereas individuals with p.(Arg307) variants lose KIF1A function [42]. Our study confirms these molecular findings; individuals with p.(Arg216His) and p.(Arg254Trp) variants had stronger language, cognitive, social responsiveness, and adaptive behaviour skills than those with p.(Arg307Gln).

Many individuals with KAND would likely benefit from AAC to support communication autonomy and continued language development in the presence of severely impaired speech and language. AAC access can reduce feelings of frustration that may be associated with challenging behaviours, whilst also fostering interactions with others [43, 44]. Clinicians should i) provide caregivers with accurate information that AAC does not hinder verbal speech development, ii) ensure that AAC allows for alternative access due to motor impairments (e.g., selecting vocabulary options with auditory, rather than solely visual, input), iii) prescribe AAC with high-contrast icons due to vision impairment, and iv) ensure that AAC access, display and vocabulary can adapt with an individual overtime [45–48].

KAND can cause progressive motor, visual and adaptive behaviour impairments, depending on the *KIF1A* pathogenic variant [3]. We reported speech and language skill regression in one-quarter of individuals but age in our cross-sectional cohort did not correlate with speech, language or adaptive behaviour. Our cohort was relatively young, limiting our ability to assess the progressive nature of the disorder. Future longitudinal studies are required to assess speech and language changes in the same individuals over time. For example, dysarthria severity may mirror fine and gross motor skill decline, as seen in other progressive neurological conditions [25, 34, 49, 50].

This study is the first to assess speech, language and AAC use in a cohort of individuals with KAND, providing insight into speech and language abilities and support needs. Our capacity to assess speech and language disorder severity, compared with overall clinical severity was limited in this study, due to its small cohort and cross-sectional nature. Future, well-powered longitudinal studies with larger participant samples could further delineate dysarthria and language disorder severity in KAND, in relation to clinical severity (e.g., cerebellar and optic never atrophy, epilepsy, gross motor impairments), and genotype.

Our assessment battery was diverse to capture the range of abilities in individuals with KAND and included both caregiver and clinician assessments. Recently, clinicians verified caregiver report of epilepsy in KAND [3]. In future, clinician assessment could further verify caregiver assessments used in this study (e.g., language ability), to account for any potential reporting biases. Additionally, caregiver assessments should use tools specifically standardised for caregivers, as used here.

Speech assessments may also prove useful in identifying response to treatment. A recent ASO study described improving dysarthria features, such as faster speech rate and improved prosody [20]. However, as systematic speech measures were not used, this interpretation should be approached cautiously. Outcome measures, such as acoustic analysis, could elucidate the presence and nature of speech improvement after therapeutic interventions.

We suggest that speech and language impairments, and co-existing medical and neurodevelopmental conditions, on a background of a progressive disease course, necessitate comprehensive and individualised speech and language intervention for individuals with KAND.

## Supplementary information

Supplemental Figure 1

Supplemental Figure 2

Supplemental Figure 3

Supplemental Figure 4

Supplementary Results

Supplemental Table 1

Supplemental Table 2

Supplemental Table 3

Supplemental Table 4

Supplemental Table 5

Supplemental Table 6

## Data Availability

The data from this study is available upon reasonable request to the corresponding author.
