# Supplementary material for: Understanding speech and language in KIF1A-associated neurological disorder
Source: Eur J Hum Genet. 2025 May 16;34(1):78–89. doi: 10.1038/s41431-025-01867-0 (PMC12816008; doi:10.1038/s41431-025-01867-0)
Supplement: Supplementary file 1 — Supplemental Figure 1 [file 41431_2025_1867_MOESM1_ESM.pdf]

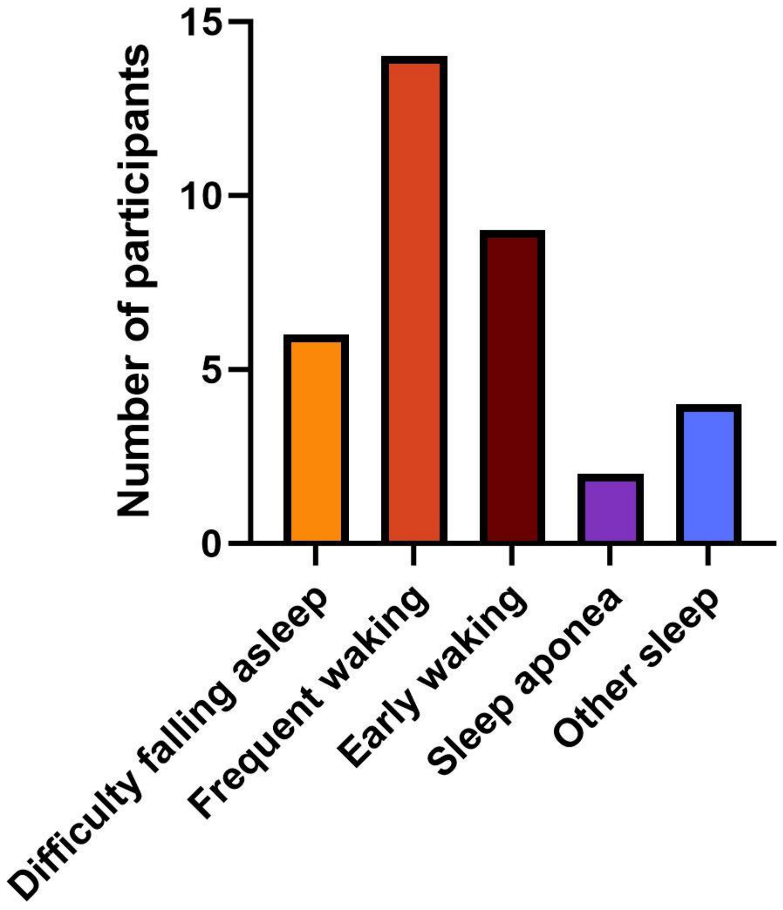

Supplemental Figure 1. Sleep disturbances in this cohort of individuals with *KIF1A*-associated neurological disorder.
Types of sleep disturbance as reported by caregivers
