# Supplementary material for: Understanding speech and language in KIF1A-associated neurological disorder
Source: Eur J Hum Genet. 2025 May 16;34(1):78–89. doi: 10.1038/s41431-025-01867-0 (PMC12816008; doi:10.1038/s41431-025-01867-0)
Supplement: Supplementary file 2 — Supplemental Figure 2 [file 41431_2025_1867_MOESM2_ESM.pdf]

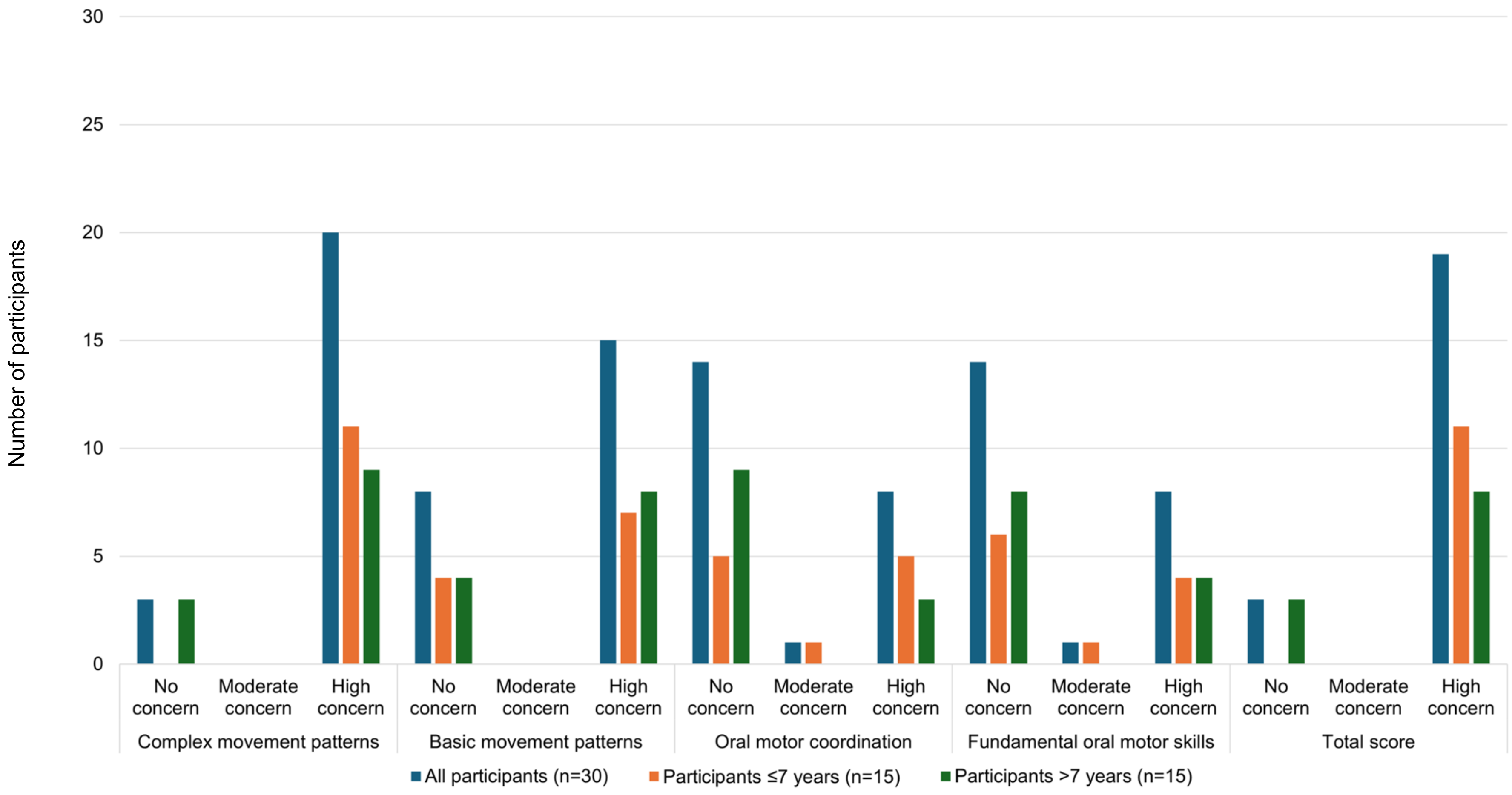

Supplemental Figure 2. Feeding skills in this cohort of individuals with *KIF1A*-associated neurological disorder. Assessed by the Child Oral Motor Proficiency Scale (n=30). No concern >10%ile, moderate concern 5-10%ile, high concern <5%ile. Normative data available for ≤7 years, participants >7 years were scored using 7-year normative data
