# Supplementary material for: Understanding speech and language in KIF1A-associated neurological disorder
Source: Eur J Hum Genet. 2025 May 16;34(1):78–89. doi: 10.1038/s41431-025-01867-0 (PMC12816008; doi:10.1038/s41431-025-01867-0)
Supplement: Supplementary file 3 — Supplemental Figure 3 [file 41431_2025_1867_MOESM3_ESM.pdf]

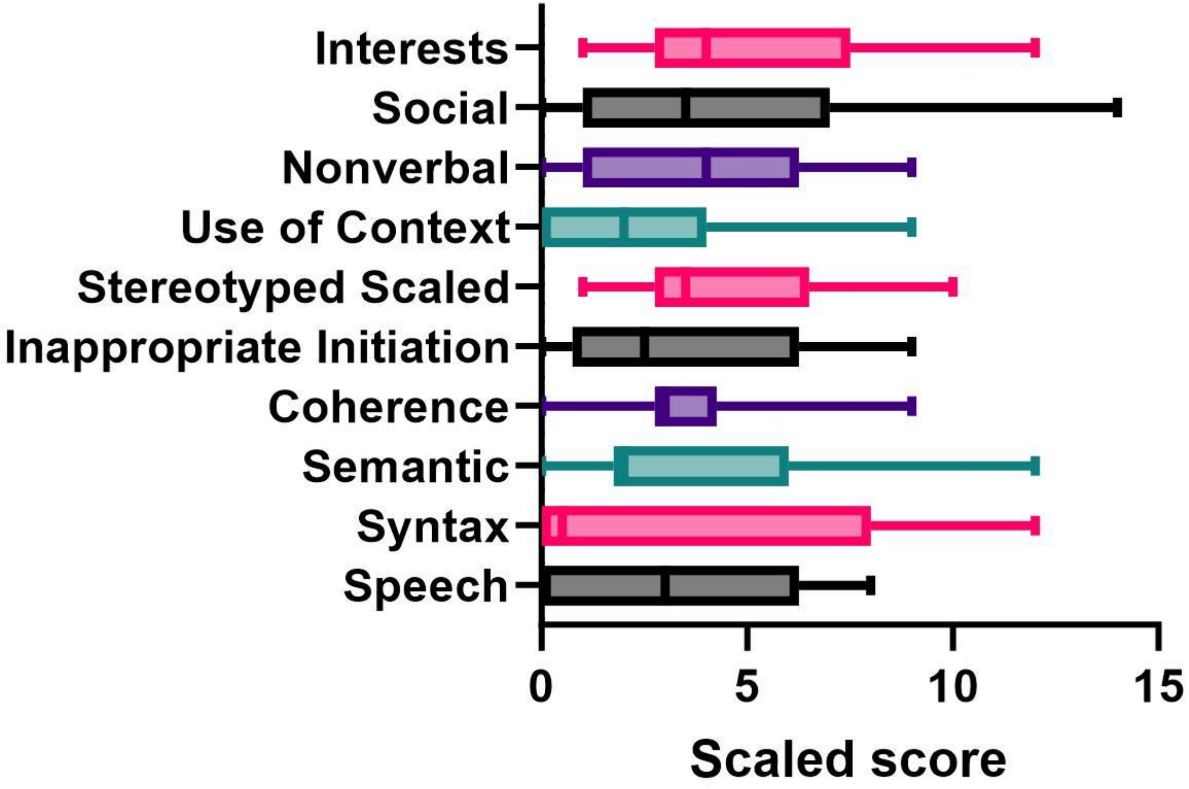

Supplemental Figure 3. Children's Communication Checklist 2nd Edition scores in 18 participants with *KIF1A*-associated neurological disorder. Normative mean=10, normative SD=3. Speech (mean=3.11, SD=3.14), syntax (mean=3.28, SD=4.24), semantic (mean=3.33, SD=3.07), coherence (mean=3.72, SD=2.19), inappropriate initiation (mean=3.39, SD=2.95), stereotyped (mean=4.67, SD=2.85), use of context (mean=2.72, SD=2.67), nonverbal (mean=3.94, SD=2.78), social (mean=4.06, SD=3.81), interests (mean=4.94, SD=3.19). There was a difference between CCC-2 scale scores ($X^2(9)=20.76$, $p=0.01$). Upper whisker=maximum, lower whisker=minimum, middle line=median.
