# Supplementary material for: Understanding speech and language in KIF1A-associated neurological disorder
Source: Eur J Hum Genet. 2025 May 16;34(1):78–89. doi: 10.1038/s41431-025-01867-0 (PMC12816008; doi:10.1038/s41431-025-01867-0)
Supplement: Supplementary file 4 — Supplemental Figure 4 [file 41431_2025_1867_MOESM4_ESM.pdf]

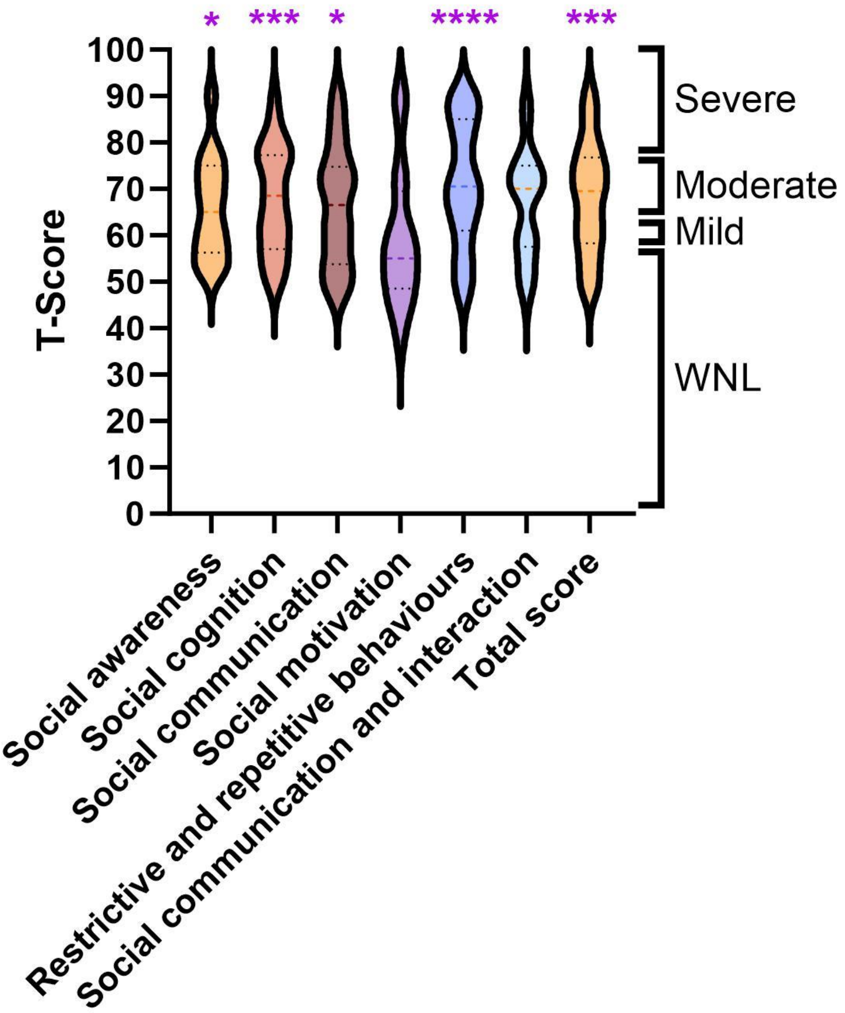

Supplemental Figure 4. Social Responsiveness Scale 2nd Edition scores in 30 participants with *KIF1A*-associated neurological disorder.

30 participants who completed the SRS-2, 15 completed the school-age form, 8 the pre-school form and 7 the adult caregiver form.

T-scores: WNL (within normal limits) <60, mild 60-65, moderate 66-75, severe >76.

Upper dotted line=3rd quartile, lower dotted line=1st quartile, dashed middle line=median. Domains: social awareness (mean=65.9, SD=10.91), social cognition (mean=67.73, SD=11.46), social communication (mean=66.43, SD=12.81), social motivation (mean=59.33, SD=14.79), restrictive and repetitive behaviours (mean=71.27, SD=13.59). Social communication and interaction: (mean=66.77, SD=11.56). Total social responsiveness score: (mean=68.2, SD=12.14). Upper dotted line=3rd quartile, lower dotted line=1st quartile, dashed middle line=median.

Purple Asterix=significant difference between social motivation and social cognition (Z=4.21, p=0.0004), social communication (Z=3.07, p=0.032), restrictive and repetitive behaviours (Z=5.18, p<0.0001), social awareness (Z=3, p=0.04) and total score (Z=4.21, p=0.0004).
