# Supplementary material for: Understanding speech and language in KIF1A-associated neurological disorder
Source: Eur J Hum Genet. 2025 May 16;34(1):78–89. doi: 10.1038/s41431-025-01867-0 (PMC12816008; doi:10.1038/s41431-025-01867-0)
Supplement: Supplementary file 5 — Supplementary Results [file 41431_2025_1867_MOESM5_ESM.pdf]

Supplementary Results. Acoustic analysis of speech in 14 individuals with *KIF1A*-associated neurological disorder

13/44 (30%) English-speaking participants with KAND completed the acoustic speech battery (7 males) ranging from 4 to 60 years of age (median age 14 years, 2 months, ranging 4 years, 8 months to 60 years, 5 months). There were two age and sex-matched control participants for each participant with KAND (n=26 controls, 14 males), ranging from 5 to 62 years of age (median age 13 years, 8 months, ranging 5 years, 8 months to 62 years, 3 months). There was not a significant difference in age between the individuals with KAND and the control group (U=166, p=0.94) (Figure 1).

Participants completed the following speech tasks; counting 1-10 (11 participants with KAND completed, 22 control participants), sustained /ɐː/ (11 participants with KAND, 22 control participants), diadochokinesis /pɐpɐpɐ/ (11 participants with KAND, 22 control participants), diadochokinesis /pɐtɐkɐ/ (10 participants with KAND, 20 control participants), and monologue (10 participants with KAND, 20 control participants). These tasks were acoustically analysed for timing, vocal tract dynamics, articulation and vocal control measures (Table 1).

Participants with KAND significantly different from controls on 23/56 (41%) measures. Counting (0/10, 0% measures significantly different) and sustained vowel (1/6, 17% measures significantly different) showed the least difference between groups. Tasks with a higher motoric load revealed greater differences between participants with KAND and controls, such as diadochokinesis (4/12, 33% /pɐpɐpɐ/ measures and 6/12, 50% /pɐtɐkɐ/ measures significantly different) and monologue

(8/16, 50% measures significantly different). For discussion of statistically significant differences between the two groups, see main manuscript Results.

Despite participants with KAND having markedly slower and more variable speech rate, the two groups did not have different voice onset times on diadochokinesis tasks, i.e., how long between the stop consonant's release (e.g., /p/) and voicing onset (e.g., /ɐ/).

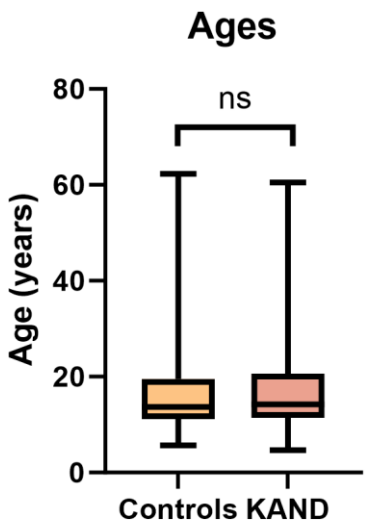

Figure 1. Ages (years) of control participants (n=26) and participant with KAND (n=13) (U=166, p=0.94).

Table 1. Acoustic speech analysis results from participants with KAND and control participants

Task	Acoustic measure	Controls		KAND		Mann-Whitney U statistic	P value	Significant
		Mean	SD	Mean	SD			
Counting 1-10	Pause mean	0.17	0.07	0.33	0.32	71	0.08	N
	Pause SD	0.09	0.04	0.24	0.27	51	0.02	N
	Pause percent	0.15	0.15	0.25	0.26	103	0.64	N
	Syllable duration mean	0.25	0.09	0.29	0.11	83	0.21	N
	Syllable duration SD	0.12	0.02	0.12	0.03	115	0.99	N
	Syllable period mean	0.34	0.14	0.51	0.43	82	0.19	N
	Syllable period SD	19	15	19	15	83	0.21	N
	Syllable per second	3.2	1.1	2.9	1.5	95	0.43	N
	Speech period	0.83	0.19	0.75	0.26	109	0.81	N
	Articulation rate	3.4	0.74	3.5	1	106	0.73	N
Sustained /ɐː/	F0 SD	18	17	19	35	93	0.29	N
	F0 mean	203	71	222	73	105	0.56	N
	MFCC1 mean	333	123	467	219	62	0.02	*
	MFCC1 SD	52	25	52	35	110	0.69	N
	MFCC2 mean	-51	97	-84	148	89	0.23	N
	MFCC2 SD	36	13	47	26	99	0.42	N
DDK /pɐpɐpɐ/	Pause mean	0.13	0.07	0.2	0.11	48	0.009	**
	Pause SD	0.06	0.04	0.18	0.1	10	<0.0001	****
	Pause percent	0.12	0.19	0.11	0.11	91	0.45	N
	Syllable duration mean	0.14	0.03	0.17	0.06	58	0.03	*
	Syllable duration SD	0.06	0.06	0.09	0.03	34	0.001	**
	Syllable period mean	0.19	0.04	0.25	0.07	35	0.001	**
	Syllable period SD	0.07	0.07	0.17	0.1	20	<0.0001	****
	Syllable per second	5.6	0.98	4.3	1.1	36	0.002	**
	Speech period	0.89	0.19	0.9	0.11	88	0.38	N
	Articulation rate	6.7	2	4.7	1.2	40	0.003	**
	VOT mean	0.03	0.007	0.03	0.01	91	0.45	N
	VOT SD	0.01	0.006	0.02	0.008	87	0.34	N
DDK /pɐtɐkɐ/	Pause mean	0.15	0.03	0.2	0.19	95	0.99	N
	Pause SD	0.08	0.03	0.11	0.06	56	0.08	N
	Pause percent	0.1	0.09	0.19	0.21	65	0.33	N
	Syllable duration mean	0.15	0.04	0.25	0.08	21	0.0003	***

	**Syllable duration SD**	0.09	0.03	0.13	0.04	41	0.01	*
	**Syllable period mean**	0.2	0.05	0.39	0.23	21	0.0003	***
	**Syllable period SD**	0.12	0.04	0.2	0.1	45	0.02	*
	**Syllable per second**	5.3	1.2	3.2	1.1	21	0.0003	***
	**Speech period**	0.91	0.09	0.83	0.21	80	0.49	N
	**Articulation rate**	5.7	1.2	3.8	1.3	24	0.0006	***
	**VOT mean**	0.04	0.009	0.04	0.02	95	0.99	N
	**VOT SD**	0.02	0.009	0.02	0.007	68	0.23	N
**Monologue**	**F0 SD**	49	13	62	23	97	0.04	*
	**F0 mean**	185	55	226	68	106	0.08	N
	**MFCC1 mean**	230	121	274	148	128	0.29	N
	**MFCC1 SD**	180	50	189	69	159	0.93	N
	**MFCC2 mean**	-8.2	86	-31	96	140	0.5	N
	**MFCC2 SD**	94	29	92	39	159	0.69	N
	**Pause mean**	0.39	0.23	0.55	0.16	62	0.001	**
	**Pause SD**	0.52	0.44	0.73	0.25	72	0.005	**
	**Syllable duration mean**	0.35	0.14	0.43	0.11	70	0.004	**
	**Syllable duration SD**	0.26	0.2	0.25	0.12	135	0.41	N
	**Syllable period mean**	0.6	0.36	0.85	0.23	51	0.0003	***
	**Syllable period SD**	0.56	0.4	0.79	0.25	72	0.005	**
	**Syllable per second**	2	0.59	1.3	0.34	51	0.0003	***
	**Speech period**	0.46	0.19	0.4	0.19	124	0.25	N
	**Speech to pause ratio**	1.1	0.73	0.96	1.2	124	0.25	N
	**Articulation rate**	4.7	1.3	3.7	1.6	73	0.005	**

*, **, ***, ****=statistically significant $p<0.05$, DDK= diadochokinesis, F0=fundamental frequency, MFCC=mel-frequency cepstral coefficient, N=No, SD=standard deviation, VOT=voice onset time.

Counting 1-10: 11 participants with KAND, 22 control participants, Sustained /ɐː/: 11 participants with KAND, 22 control participants, DDK /pɐpɐpɐ/: 11 participants with KAND, 22 control participants, DDK /pɐtɐkɐ/: 10 participants with KAND, 20 control participants, Monologue: 10 participants with KAND, 20 control participants.
