# Supplementary material for: Understanding speech and language in KIF1A-associated neurological disorder
Source: Eur J Hum Genet. 2025 May 16;34(1):78–89. doi: 10.1038/s41431-025-01867-0 (PMC12816008; doi:10.1038/s41431-025-01867-0)
Supplement: Supplementary file 6 — Supplemental Table 1 [file 41431_2025_1867_MOESM6_ESM.pdf]

Supplemental Table 1. Acoustic speech battery and measures

Task	Speech subsystem assessed	Outcomes
Monologue	Prosody	Mean and variation in silence length, mean and variation in syllable length, syllables per second, articulation rate
	Phonation	Fundamental frequency variability
	Articulation	Mel Frequency Cepstral Coefficient 1 & 2
Prolonged ‘ah’ (2 attempts)	Phonation	Fundamental frequency, Mel Frequency Cepstral Coefficient 1 & 2
Counting 1-10 (2 attempts)	Prosody	Mean and variation in silence length, mean and variation in syllable length, syllables per second, articulation rate
Diadochokinetic Alternating Motion Rate “papapa” (2 attempts)	Articulation	Voice onset time
	Prosody	Mean and variation in silence length, mean and variation in syllable length, syllables per second, articulation rate
Diadochokinetic Sequential Motion Rate “pataka” (2 attempts)	Articulation	Voice onset time
	Prosody	Mean and variation in silence length, mean and variation in syllable length, syllables per second, articulation rate

Data collection and analysis using Redenlab’s ® Analyze pipeline.
