# Supplementary material for: Understanding speech and language in KIF1A-associated neurological disorder
Source: Eur J Hum Genet. 2025 May 16;34(1):78–89. doi: 10.1038/s41431-025-01867-0 (PMC12816008; doi:10.1038/s41431-025-01867-0)
Supplement: Supplementary file 7 — Supplemental Table 2 [file 41431_2025_1867_MOESM7_ESM.pdf]

Supplemental Table 2. Genotype of 44 individuals with *KIF1A*-associated neurological disorder

Participant ID	Age at assessment (range, yrs)	cDNA change (NM_001244008.2)	Protein change (NP_001230937.1)	Genomic change (GRCh38)	Inheritance†	Type	Age diagnosed (years)
1	11-12	c.32G>A	p.(Arg11Gln)	g.240797721C>T	De novo	Missense	7
2	39-40	c.32G>A	p.(Arg11Gln)	g.240797721C>T	Unknown	Missense	21
3	17-18	c.32G>A	p.(Arg11Gln)	g.240797721C>T	De novo	Missense	14
4	11-12	c.289G>C	p.(Gly97Arg)	g.240788125C>G	De novo	Missense	9
5*	1-2	c.296C>A	p.(Thr99Lys)	g.240788118G>T	De novo	Missense	0.5
6	11-12	c.296C>T	p.(Thr99Met)	g.240788118G>A	De novo	Missense	6
7	3-4	c.304G>A	p.(Gly102Ser)	g.240788110C>T	De novo	Missense	3
8	13-14	c.304G>A	p.(Gly102Ser)	g.240788110C>T	De novo	Missense	7
9	23-24	c.308A>C	p.(Lys103Thr)	g.240788106T>G	De novo	Missense	17
10	15-16	c.350G>T	p.(Gly117Val)	g.240788064C>A	De novo	Missense	7
11	1-2	c.443A>G	p.(Glu148Gly)	g.240786500T>C	De novo	Missense	1
12	23-24	c.443A>T	p.(Glu148Val)	g.240786500T>A	Paternal mosaic	Missense	21
13	21-32	c.470T>A	p.(Leu157His)	g.240786473A>T	Unknown	Missense	26
14	13-14	c.506G>A	p.(Arg169Lys)	g.240786437C>T	De novo	Missense	5
15	1-2	c.596G>A	p.(Gly199Glu)	g.240786347C>T	De novo	Missense	1

16	1-2	c.604G>C	p.(Ala202Pro)	g.240786339C>G	Unknown	Missense	1
17	1-2	c.641G>A	p.(Ser214Asn)	g.240785068C>T	De novo	Missense	1
18	11-12	c.647G>A	p.(Arg216His)	g.240785062C>T	De novo	Missense	3
19	3-4	c.647G>A	p.(Arg216His)	g.240785062C>T	Unknown	Missense	3
20	3-4	c.647G>A	p.(Arg216His)	g.240785062C>T	De novo	Missense	2
21	3-4	c.746T>C	p.(Leu249Pro)	g.240783791A>G	Unknown	Missense	1
22	23-24	c.746T>C	p.(Leu249Pro)	g.240783791A>G	De novo	Missense	20
23	59-60	c.761G>A	p.(Arg254Gln)	g.240783776C>T	Unknown	Missense	58
24	5-6	c.760C>T	p.(Arg254Trp)	g.240783777G>A	De novo	Missense	4
25	3-4	c.760C>T	p.(Arg254Trp)	g.240783777G>A	De novo	Missense	1
26	3-4	c.760C>T	p.(Arg254Trp)	g.240783777G>A	Unknown	Missense	2
27	3-4	c.812T>C	p.(Ile271Thr)	g.240783096A>G	De novo	Missense	2
28	19-20	c.839A>G	p.(Lys280Arg)	g.240783069T>C	De novo	Missense	15
29	7-8	c.920G>A	p.(Arg307Gln)	g.240775889C>T	De novo	Missense	4
30	3-4	c.920G>A	p.(Arg307Gln)	g.240775889C>T	De novo	Missense	1
31	7-8	c.920G>A	p.(Arg307Gln)	g.240775889C>T	De novo	Missense	1
32	1-2	c.920G>A	p.(Arg307Gln)	g.240775889C>T	De novo	Missense	1
33	5-6	c.946C>T	p.(Arg316Trp)	g.240775863G>A	Unknown	Missense	4

34	11-12	c.946C>T	p.(Arg316Trp)	g.240775863G>A	Unknown	Missense	4
35	11-12	c.946C>T	p.(Arg316Trp)	g.240775863G>A	Unknown	Missense	8
36	3-4	c.946C>T	p.(Arg316Trp)	g.240775863G>A	Unknown	Missense	2
37	3-4	c.946C>T	p.(Arg316Trp)	g.240775863G>A	De novo	Missense	3
38	13-14	c.946C>T	p.(Arg316Trp)	g.240775863G>A	De novo	Missense	10
39	3-4	c.946C>T	p.(Arg316Trp)	g.240775863G>A	De novo	Missense	2
40	9-10	c.976G>C	p.(Ala326Pro)	g.240774244C>G	De novo	Missense	8
41	7-8	c.2012_2013delinsCC	p.(Gln671Pro)	g.240763028_240763029delinsGG	De novo	Missense	7
42	9-10	c.146_166del	p.(Ser49_Tyr56delinsAsn)	g.240789253_240789273del	Unknown	Deletion	4
43	17-18	c.146_166del	p.(Ser49_Tyr56delinsAsn)	g.240789253_240789273del	Paternal mosaic	Deletion	11
44	3-4	c.1038-1G>A			De novo	Splice acceptor variant	2

*=Deceased, †=all heterozygous, and pathogenic or likely pathogenic, Yrs=years
