# Supplementary material for: Understanding speech and language in KIF1A-associated neurological disorder
Source: Eur J Hum Genet. 2025 May 16;34(1):78–89. doi: 10.1038/s41431-025-01867-0 (PMC12816008; doi:10.1038/s41431-025-01867-0)
Supplement: Supplementary file 8 — Supplemental Table 3 [file 41431_2025_1867_MOESM8_ESM.pdf]

Supplemental Table 3. Motor skills, neurodevelopmental and neurological features, and vision impairment in 44 individuals with *KIF1A*-associated neurological disorder

Participant	Motor				Neurodevelopmental conditions				Neurological features			
ID	Sit without support (mo)	Crawling (mo)	Walking independently (mo)	Current walking ability	Cognition*	Autism	ADHD	Behavioural problems	Epilepsy	MRI findings	Sleep disturbance	Visual impairment
1	≥13	≥14	≥16	Walks typically	Average	-	+	+	-	-	-	+
2	4-7	7-10	≥16	Limited distance	Mild	+	-	+	+	+	+	-
3	4-7	7-10	9-12	Unsteady gait	Mild	+	-	-	-	-	+	-
4	8-10	11-13	≥16	Walks typically	Mild	-	+	-	-	-	-	+
5	NYA	NYA	NYA	Does not walk	Severe	-	-	-	+	+	+	+
6	NYA	NYA	NYA	Does not walk	Severe	-	+	-	+	+	-	+
7	≥13	≥14	NYA	Walk with cane or other aid	NA	+	+	+	-	+	-	+
8	8-10	11-13	≥16	Walks typically	Mild	+	+	+	-	-	+	+

9	11-12	11-13	≥16	Walk with cane or other aid	Severe	-	+	+	-	+	+	+
10	≥13	≥14	≥16	Unsteady gait	Severe	+	+	-	-	+	-	+
11	NYA	NYA	NYA	Does not walk	Severe	-	-	+	-	-	+	+
12	≥13	≥14	NYA	Limited distance	Severe	+	-	+	+	+	-	+
13	≥13	≥14	NYA	Does not walk	Severe	-	-	-	+	+	-	+
14	4-7	7-10	13-15	Limited distance	Moderate	-	-	+	-	NA	+	-
15	NYA	NYA	NYA	Does not walk	NA	-	-	-	+	+	-	+
16	NYA	NYA	NYA	Does not walk	NA	-	-	-	+	+	-	+
17	8-10	11-13	NYA	Does not walk	Mild	-	-	-	+	+	+	+
18	4-7	7-10	≥16	Walk with cane or other aid	Mild	-	-	-	-	+	-	+
19	8-10	11-13	NYA	Limited distance	NA	-	-	-	-	+	+	+
20	8-10	7-10	≥16	Unsteady gait	NA	-	-	-	-	+	+	+
21	≥13	NYA	NYA	Does not walk	NA	-	-	-	+	+	+	+
22	≥13	NYA	NYA	Does not walk	Moderate	-	-	+	+	+	+	+
23	≥13	≥14	≥16	Stopped walking	Moderate	-	-	+	-	-	+	+

24	4-7	11-13	NYA	Walk with cane or other aid	Severe	-	-	+	+	+	-	+
25	≥13	11-13	NYA	Does not walk	Mild	-	-	-	+	+	+	+
26	8-10	11-13	NYA	Walk with cane or other aid	Borderline	-	-	-	-	+	-	+
27	NYA	NYA	NYA	Does not walk	Severe	-	-	-	+	-	+	+
28	11-12	≥14	≥16	Walk with cane or other aid	Mild	-	-	-	+	+	+	+
29	NYA	NYA	NYA	Does not walk	NA	-	-	-	-	+	+	+
30	≥13	≥14	NYA	Does not walk	Severe	-	-	-	-	-	-	+
31	≥13	≥14	NYA	Does not walk	Moderate	+	-	+	+	+	-	+
32	NYA	NYA	NYA	Does not walk	Moderate	-	-	-	-	+	+	+
33	11-12	≥14	NYA	Walk with cane or other aid	NA	+	-	-	-	+	+	+
34	NYA	11-13	NYA	Walk with cane or other aid	Moderate	+	+	+	-	+	+	+
35	≥13	≥14	NYA	Does not walk	Mild	+	+	-	-	+	+	+

36	8-10	11-13	NYA	Walk with cane or other aid	NA	-	-	-	-	-	-	+
37	11-12	7-10	NYA	Does not walk	NA	-	-	-	+	+	-	+
38	≥13	≥14	NYA	Does not walk	Moderate	-	+	+	-	+	-	+
39	≥13	≥14	NYA	Limited distance	NA	-	-	-	-	+	+	+
40	11-12	≥14	≥16	Unsteady gait	Mild	-	+	-	-	-	-	-
41	≥13	≥14	NYA	Walk with cane or other aid	Moderate	-	-	-	-	+	-	+
42	11-12	11-13	≥16	Unsteady gait	Severe	+	+	+	-	+	-	+
43	11-12	11-13	≥16	Unsteady gait	Severe	+	-	-	-	+	+	+
44	8-10	≥14	≥16	Walks typically	Moderate	-	-	+	-	-	+	+

+=feature present, -=feature absent, *=Intelligence quotient (IQ): Average (>85), Borderline (70-84), Mild (50-70), Moderate (35-50), Severe (<35), ADHD=Attention Deficit Hyperactivity Disorder, Mo=months, MRI=Magnetic Resonance Imaging, NA=Not assessed, NYA=Not achieved, TY=too young
