# Supplementary material for: Understanding speech and language in KIF1A-associated neurological disorder
Source: Eur J Hum Genet. 2025 May 16;34(1):78–89. doi: 10.1038/s41431-025-01867-0 (PMC12816008; doi:10.1038/s41431-025-01867-0)
Supplement: Supplementary file 9 — Supplemental Table 4 [file 41431_2025_1867_MOESM9_ESM.pdf]

**Supplemental Table 4. Education and therapy in 44 individuals with *KIF1A*-associated neurological disorder**

| Participant ID | Speech therapy | | | | | | | Other therapies & school | | | | Interests & strengths | | | | | | Dislikes |
|---|---|---|---|---|---|---|---|---|---|---|---|---|---|---|---|---|---|---|
| | SP | AAC | Sign/ gesture | Sign level use | Aided AAC | AAC system used | Aided AC level use | OT | PT | Current activity | Highest schooling level | Music | Social | Humour | Determined | Swim | Games | Noise |
| 1 | + | + | + | Natural Gestures, Single word signs | - | - | - | + | + | School | Mainstream primary school | + | + | - | - | - | + | + |
| 2 | + | - | - | - | - | - | - | + | + | <30 hours per week part-time work | Diploma | - | + | - | + | - | + | - |
| 3 | + | - | - | - | - | - | - | + | + | Part-time school, part-time homeschool | Mainstream secondary school | - | + | + | + | + | + | - |
| 4 | + | - | - | - | - | - | - | + | - | School | Mainstream primary school | + | - | - | - | + | + | - |
| 5 | - | - | - | - | - | - | - | - | - | Passed away | TY | NA | NA | NA | NA | NA | NA | NA |
| 6 | + | + | - | - | - | - | - | + | + | School | Mainstream primary school | NA | NA | NA | NA | NA | NA | NA |
| 7 | + | + | - | - | + | TDSnap | NR | + | + | Daycare/Kindergarten | Specialised daycare | NA | NA | NA | NA | NA | NA | NA |
| 8 | + | - | - | - | | | | + | + | School | Mainstream secondary school | - | + | + | + | - | + | - |
| 9 | + | + | + | Natural Gestures | - | | | + | + | Disability day program | Diploma | - | + | + | + | - | + | + |
| 10 | + | - | | | | | | + | + | School | Specialised secondary school | - | + | - | - | - | + | - |
| 11 | + | - | | | | | | + | + | TY | Mainstream daycare/kindergarten | NA | NA | NA | NA | NA | NA | NA |
| 12 | + | + | + | Natural Gestures, Single word signs | + | Previously, communication books, iPad applications | Combining 2-3 symbols together | + | + | Disability day program | Specialised secondary school | + | + | - | + | - | + | + |
| 13 | - | + | - | - | - | - | - | + | + | Disability day program | TY | + | + | - | - | - | - | - |

| | | | | | | | | | | | | | | | | | | |
|---|---|---|---|---|---|---|---|---|---|---|---|---|---|---|---|---|---|---|
| 14 | - | - | - | - | - | - | - | + | + | Homeschool | Home schooling | - | - | - | + | - | + | - |
| 15 | - | - | - | - | - | - | - | + | + | TY | TY | + | + | - | - | - | - | + |
| 16 | - | - | - | - | - | - | - | + | + | Daycare/Kindergarten | Mainstream daycare/kindergarten | + | + | - | - | - | - | - |
| 17 | + | - | - | - | - | - | - | + | + | TY | TY | NA | NA | NA | NA | NA | NA | NA |
| 18 | + | + | - | - | + | Trialled Proloquo2Go | Single symbols | + | + | School | Mainstream primary school | - | + | + | - | - | + | - |
| 19 | + | + | + | Natural Gestures, Single word signs | - | - | - | - | + | TY | Mainstream daycare/kindergarten | - | + | + | - | - | + | - |
| 20 | + | - | + | Natural Gestures, Single word signs | - | - | - | + | + | Daycare/Kindergarten | Specialised daycare/kindergarten | - | - | - | + | - | - | + |
| 21 | + | + | - | - | - | - | - | + | + | Daycare/Kindergarten | Specialised daycare/kindergarten | + | - | - | + | + | - | + |
| 22 | + | + | + | Natural Gestures, Single word signs | - | - | - | + | + | Daycare/Kindergarten | Specialised secondary school | - | + | + | - | - | + | - |
| 23 | - | - | - | - | - | - | - | + | + | Disability day program | Specialised secondary school | - | + | - | - | - | - | - |
| 24 | + | + | - | - | - | - | - | + | + | School | Mainstream kindergarten/preschool | - | + | - | + | - | + | - |
| 25 | + | + | - | - | - | - | - | + | + | Daycare/Kindergarten | Specialised daycare/kindergarten | + | + | - | + | - | + | - |
| 26 | + | + | - | - | + | Trialled iPad AAC application | Single symbols | + | + | Daycare/Kindergarten | Specialised childcare | + | - | + | + | + | + | + |
| 27 | + | - | - | - | - | - | - | + | + | At home | TY | + | - | + | - | - | + | - |
| 28 | + | - | - | - | - | - | - | + | + | School | Specialised secondary school | NA | NA | NA | NA | NA | NA | NA |
| 29 | + | + | - | - | - | - | - | - | + | School | Mainstream primary school | NA | NA | NA | NA | NA | NA | NA |

| | | | | | | | | | | | | | | | | | | |
|---|---|---|---|---|---|---|---|---|---|---|---|---|---|---|---|---|---|---|
| 30 | + | + | + | Not using independently | - | - | - | + | + | Specialised centre | Specialised daycare/kindergarten | NA | NA | NA | NA | NA | NA | NA |
| 31 | + | - | - | - | - | - | - | + | + | School | Specialised daycare/kindergarten | + | + | + | - | + | - | + |
| 32 | + | - | - | - | - | - | - | + | + | TY | TY | NA | NA | NA | NA | NA | NA | NA |
| 33 | + | + | + | Natural Gestures, Single word signs | + | PODD book: 12 cells high contrast | Combining 2-3 symbols together | + | + | School | Specialised primary school | + | + | + | - | - | - | - |
| 34 | + | + | + | Natural Gestures | + | Picture system | Single symbols | + | + | School | Mainstream daycare/kindergarten | - | - | - | - | - | + | - |
| 35 | + | - | - | - | - | - | - | + | + | School | Mainstream primary school | + | + | - | - | - | - | + |
| 36 | + | + | - | - | - | - | - | + | + | Daycare/Kindergarten | Specialised daycare/kindergarten | - | + | - | + | + | + | - |
| 37 | + | + | - | - | + | - | - | + | + | School | Specialised daycare/kindergarten | NA | NA | NA | NA | NA | NA | NA |
| 38 | - | + | - | - | - | - | - | + | + | School | Mainstream secondary school | - | + | + | - | - | - | - |
| 39 | + | - | - | - | - | - | - | + | + | Daycare/Kindergarten | Mainstream daycare/kindergarten | + | + | - | + | - | - | + |
| 40 | + | + | + | - | + | - | - | + | + | School | Specialised primary school | - | + | - | - | - | + | + |
| 41 | + | - | - | - | - | - | - | + | + | School | Mainstream primary school | + | + | + | + | + | + | + |
| 42 | + | - | - | - | - | - | - | + | + | School | Specialised primary school | NA | NA | NA | NA | NA | NA | NA |
| 43 | + | + | - | - | + | Communication book | Not defined | + | + | School | Specialised primary school | NA | NA | NA | NA | NA | NA | NA |
| 44 | + | - | - | - | - | - | - | + | + | Daycare/Kindergarten | Mainstream daycare/kindergarten | NA | NA | NA | NA | NA | NA | NA |

+=feature present, -=not reported, AAC= augmentative and alternative communication, Mo=Months, NA=not assessed, NR=Not reported, PODD=Pragmatic Organisation Dynamic Display, SP=Speech pathology, TY=Too young for school/daycare, Yrs=Years
