# Supplementary material for: Understanding speech and language in KIF1A-associated neurological disorder
Source: Eur J Hum Genet. 2025 May 16;34(1):78–89. doi: 10.1038/s41431-025-01867-0 (PMC12816008; doi:10.1038/s41431-025-01867-0)
Supplement: Supplementary file 10 — Supplemental Table 5 [file 41431_2025_1867_MOESM10_ESM.pdf]

Supplemental Table 5. Medical features in 44 individuals with *KIF1A*-associated neurological disorder

Participant ID	Sex	Skill loss					Muscular		MRI findings	Vision									Skin/Teeth			Other health conditions
		Speech/language loss	Fine motor skills loss	Gross motor skills loss	Play skills loss	Other skill loss	Tremor	Hypotonia		Hypermetropia	Myopia	Squint	Strabismus	Nystagmus	CVI	Exotropia	Optic nerve atrophy	Other vision impairment	Skin conditions	Thermal reg difficulties	Dental problems	
**1**	M	-	+	+	-	+	-	+	-	-	-	-	-	-	+	+	-	-	-	+	-	Asthma, food allergy, frequent diarrhoea
**2**	F	-	-	+	-	-	-	-	Unspecified	-	-	-	-	-	-	-	-	-	Seborrheic dermatitis	-	-	Chronic pain, reflux
**3**	M	-	-	-	-	-	-	+	-	-	-	-	-	-	-	-	-	-	-	-	-	Food allergy, frequent diarrhoea
**4**	M	-	-	-	-	-	-	-	-	+	-	-	-	-	-	+	-	-	Eczema	NA	NA	-
**5**	M	+	+	+	+	+	-	+	Thin corpus callosum	-	-	-	-	+	+	-	-	-	Eczema, dry/itchy skin	+	TY	Chronic pain
**6**	M	NA	NA	NA	NA	-	-	NA	Hypoplasia of the corpus callosum, white matter reduction	-	-	-	-	+	-	-	-	Unspecified	-	NA	-	Constipation
**7**	M	NA	NA	NA	NA	-	+	NA	Microcephaly, cerebellar atrophy with widening of sulci and 4th ventricle, hypotrophy corpus callosum, agenesis of	-	-	-	-	-	-	-	-	Unspecified	-	NA	-	Constipation

									anterior white commissure and interregional commissure, absence of notable parenchymal signal abnormality													
**8**	M	-	-	-	-	-	Hands	+	-	+	+	-	+	-	-	-	-	-	-	-	-	Anxiety
**9**	M	-	-	+	-	-	-	+	Delayed myelination	-	+	-	-	-	-	-	+	Two different sized pupils, physiologic anisocoria	-	-	-	Anxiety
**10**	M	-	-	+	-	-	-	+	Cerebral atrophy	-	-	-	-	-	-	-	+	-	-	+	-	-
**11**	M	NA	NA	NA	NA	-	+	NA	-	+	+	-	-	-	-	-	-	+	-	NA	TY	-
**12**	M	-	-	+	-	-	-	+	Over several MRIs: progressive bilateral cerebellar hemisphere and vermis atrophy, bilateral optic atrophy& diminutive optic chiasm, enlargement of the cisterna magna, hypoplastic right vertebral artery V4 segment	-	-	-	+	+	+	-	-	-	-	+	Small jaw, complex orthodontics	Seasonal allergy, Recurrent UTIs, frequent diarrhoea, constipation
**13**	M	+	-	-	-	+	-	+	Atrophy of vermis inferior, wide ventricles	-	-	-	-	+	-	-	+	Blind	-	-	-	Constipation
**14**	M	-	-	-	-	-	-	-	NA	-	-	-	-	-	-	-	-	-	-	-	Frequent dental caries	-
**15**	F	+	-	-	-	-	-	+	Cerebellar atrophy,	-	-	-	-	-	+	-	-	-	Eczema, dry/itchy skin	+	-	Duplex kidney and

									prominence of the folia													no control of bladder
**16**	M	-	-	-	-	-	-	+	White matter lesions	-	-	-	-	-	-	-	+	-	-	-	-	-
**17**	F	NA	NA	NA	NA	+	+	NA	Thinning of corpus callosum mainly posterior aspect, smaller vol of regional white matter, focal dilation of the atria.	-	-	+	-	-	-	-	-	Unspecified	-	NA	-	-
**18**	F	-	-	-	-	-	-	+	Cerebella atrophy, pituitary gland cyst found	-	+	-	-	+	-	-	-	-	-	+	-	-
**19**	M	-	-	-	-	-	-	+	Scar tissue though nil concern	-	-	-	+	+	-	-	-	Double elevator palsy	-	-	TY	Constipation
**20**	F	-	-	-	-	-	-	+	Abnormal sulcation and mild cortical thickening in the right postcentral area, cerebellar volume reduction	-	-	-	+	-	-	-	-	-	Eczema	+	-	Food allergy
**21**	F	+	-	-	-	-	+	+	Insufficient myelination of the optic radiations	-	-	-	-	-	+	-	+	-	Eczema	-	-	Reflux
**22**	F	-	-	-	-	-	+	+	Left mesial temporal sclerosis, cerebella atrophy, negative spectroscopy	-	-	-	-	-	-	-	+	Cortical inattentiveness, pallor optic nerve pallor.	-	+	Frequent dental caries, complex orthodontics	Bilateral partially duplicated kidneys, frequent UTI's and a kidney infection, 1st degree bladder reflux, oesophageal reflux, constipation

**23**	M	+	+	+	-	-	Hands	-	Cerebellar atrophy, cerebellar vermis atrophy, hyperintensity of cerebral white matter on MRI	-	+	+	-	-	-	-	-	-	-	-	Frequent dental caries	Rapid cycle bi-polar disorder, neurological incontinence, arthritis in hands, diabetes, frequent diarrhoea
**24**	M	-	-	+	-	-	Hands	-	Progressive atrophy of vermis, mild atrophy of superior aspect of bilateral cerebellar hemispheres, macrocephaly	+	-	-	-	+	+	-	-	-	-	-	Staining	-
**25**	F	-	-	+	-	-	Hands	+	Cerebellar atrophy	-	-	-	-	+	+	-	-	Astigmatism	-	+	TY	-
**26**	M	-	-	-	-	-	-	+	Difference in the corpus collosum, differing size of the splenium and genu	-	-	-	-	-	-	-	+	-	-	-	-	Reflux
**27**	F	-	+	-	-	-	-	+	-	-	-	-	-	-	+	-	-	-	-	+	-	Constipation
**28**	M	NA	NA	NA	NA	+	-	NA	Ventriculomegaly, abnormal cerebellum	-	+	-	+	-	-	-	-	-	-	NA	-	Medication allergy, neurogenic bladder, frequent diarrhoea
**29**	F	NA	NA	NA	NA	-	-	NA	Cerebellum hypoplasia, corpus callosum hypoplasia	-	-	-	-	+	-	-	+	-	-	NA	-	Reflux
**30**	F	NA	NA	NA	NA	-	-	NA	-	+	-	-	-	+	-	-	-	Unspecified	-	NA	-	-
**31**	F	-	-	-	-	-	Hands	+	Cerebellar atrophy with thinned corpus callosum and	-	-	-	-	-	+	-	-	-	Eczema, dry/itchy skin	-	-	Frequent diarrhoea

									increased periventricular white matter T2 signal primarily posteriorly, asymmetrical prominence of the posterior aspect of the R gyrus rectus, hypoplastic optic nerves and optic chiasm													
**32**	F	NA	NA	NA	NA	+	+	NA	Brain MRI showing multiple periventricular hyperintensity foci in both lateral ventricles without ventricular dilation or alteration in morphology	+	-	-	-	-	-	-	-	Unspecified	-	NA	-	Constipation
**33**	F	+	-	-	-	-	-	+	Cerebellar atrophy	-	-	-	-	-	+	-	-	-	Eczema	-	-	Constipation
**34**	M	-	-	+	-	-	-	+	Intracranial hypotension	-	-	-	-	-	-	-	+	-	Eczema	+	+	Anxiety, asthma, food allergy
**35**	F	-	-	-	-	-	Hands	+	Cerebellar atrophy	-	+	-	+	+	+	-	-	-	-	+	+	Abdominal migraines, reflux
**36**	F	-	-	-	-	-	-	+	-	+	-	-	+	+	-	-	+	-	-	+	-	Medication allergy
**37**	M	NA	NA	NA	NA	-	-	NA	Optic nerve atrophy	-	-	-	-	-	-	-	-	Unspecified	-	NA	-	-
**38**	M	-	-	-	-	-	-	+	Cerebellum atrophy	-	-	-	-	-	-	-	+	-	-	-	Complex orthodontics	-
**39**	F	+	-	-	-	-	-	+	Bilateral optic pallor, slight thinning of the optic chiasm	-	+	-	+	+	-	-	-	-	Eczema	-	TY	Constipation
**40**	F	-	-	-	-	-	+	-	-	-	-	-	-	-	-	-	-	-	-	-	-	-

**41**	F	-	-	-	-	-	Hands	+	Optic nerve atrophy, progressive cerebellar atrophy	-	+	-	+	+	-	-	+	-	Eczema	-	-	-
**42**	M	NA	NA	NA	NA	-	+	NA	Discreet widening of the upper vermian fissures	+	-	-	+	+	-	-	-	-	Face spots as skin does not tan	NA	-	-
**43**	M	NA	NA	NA	NA	-	+	NA	Progressive atrophy of the cerebral vermis and hemispheres	+	-	-	+	+	-	-	-	Unspecified	-	NA	-	Anxiety
**44**	M	NA	NA	NA	NA	-	-	+	-	-	+	-	+	-	-	-	-	-	-	NA	-	-

+=Feature present, -=feature absent, CVI=cortical visual impairment, F=Female, M=Male, Mo=Months, MRI=Magnetic resonance imaging, NA=Not assessed, Reg=Regulation, TY=Too young, Yrs=Years
