# Supplementary material for: Understanding speech and language in KIF1A-associated neurological disorder
Source: Eur J Hum Genet. 2025 May 16;34(1):78–89. doi: 10.1038/s41431-025-01867-0 (PMC12816008; doi:10.1038/s41431-025-01867-0)
Supplement: Supplementary file 11 — Supplemental Table 6 [file 41431_2025_1867_MOESM11_ESM.pdf]

Supplemental Table 6. Epilepsy in 44 individuals with *KIF1A*-associated neurological disorder

ID	EEG abnormalities	Epilepsy	Seizure types	Seizure frequency	Antiseizure medication	Seizures managed by antiseizure medication
1	+	-	NA	NA	NA	NA
2	-	+	Generalised tonic clonic, absence	Once	Lamotrigine	+
3	-	-	NA	NA	NA	NA
4	-	-	NA	NA	NA	NA
5	+	+	Not specified	10-15 per day	Levetiracetam, Oxcarbazepine, Vigabatrin, Valproate, Clobazam	+
6	+	+	Unknown	Sporadic	Levetiracetam	+
7	-	-	NA	NA	NA	NA
8	-	-	NA	NA	NA	NA
9	-	-	NA	NA	NA	NA
10	-	-	NA	NA	NA	NA
11	-	-	NA	NA	NA	NA
12	+	+	Febrile, grandmal, atonic, myoclonic, absence	Daily, frequency dependent on seizure type	Levetiracetam, Divalproex Sodium, Cannabidiol	+
13	+	+	Grandmal, absence	>20 Grandmal, management increasingly difficult	Levetiracetam, Lamotrigine, Sulthiame	+
14	-	-	NA	NA	NA	NA
15	+	+	Unknown	Irregular, increasing in frequency	Levetiracetam	+
16	+	+	Complex febrile seizure	Once, admitted to neonatal intensive care unit	-	-
17	+	+	Absence	Not reported	Levetiracetam	+
18	-	-	NA	NA	NA	NA
19	-	-	NA	NA	NA	NA
20	+	-	NA	NA	NA	NA
21	+	+	Unknown	No seizures, just abnormal EEG	Clobazam	+
22	+	+	Focal marching, status epilepticus	1 per month, increasing in frequency	Levetiracetam, Lacosamide, Zonisamide, Cannabidiol	+
23	-	-	NA	NA	NA	NA
24	+	+	Grand mal, absence	Once	Levetiracetam, Lorazepam	+
25	+	+	Unknown	No active seizures, has ‘staring spells’	Clobazam, Levetiracetam	+
26	-	-	NA	NA	NA	NA

**27**	+	+	Focal, absence	8-15 per day	Levetiracetam	+
**28**	+	+	Status epilepticus	Once	Levetiracetam	+
**29**	+	-	NA	NA	NA	NA
**30**	+	-	NA	NA	NA	NA
**31**	+	+	Focal	1 every few months	Levetiracetam, Cannabidiol, Valium	+
**32**	-	-	NA	NA	NA	NA
**33**	-	-	NA	NA	NA	NA
**34**	-	-	NA	NA	NA	NA
**35**	+	-	NA	NA	NA	NA
**36**	-	-	NA	NA	NA	NA
**37**	+	+	Generalised	Frequent, at night	Levetiracetam	+
**38**	-	-	NA	NA	NA	NA
**39**	-	-	NA	NA	NA	NA
**40**	-	-	NA	NA	NA	NA
**41**	-	-	NA	NA	NA	NA
**42**	+	-	NA	NA	NA	NA
**43**	-	-	NA	NA	NA	NA
**44**	+	-	NA	NA	NA	NA

+=feature present, -=feature absent, EEG=Electroencephalogram
